# Assessment of the quality of measures of child oral health-related quality of life

**DOI:** 10.1186/1472-6831-14-40

**Published:** 2014-04-23

**Authors:** Fiona Gilchrist, Helen Rodd, Chris Deery, Zoe Marshman

**Affiliations:** 1 Unit of Oral Health and Development, School of Clinical Dentistry, Sheffield S10 2TA, UK

## Abstract

**Background:**

Several measures of oral health-related quality of life have been developed for children. The most frequently used are the Child Perceptions Questionnaire (CPQ), the Child Oral Impacts on Daily Performances (C-OIDP) and the Child Oral Health Impact Profile (COHIP). The aim of this study was to assess the methodological quality of the development and testing of these three measures.

**Methods:**

A systematic search strategy was used to identify eligible studies published up to December 2012, using both MEDLINE and Web of Science. Titles and abstracts were read independently by two investigators and full papers retrieved where the inclusion criteria were met. Data were extracted by two teams of two investigators using a piloted protocol. The data were used to describe the development of the measures and their use against existing criteria. The methodological quality and measurement properties of the measures were assessed using standards proposed by the Consensus-based Standards for the Selection of Health Measurement Instruments (COSMIN) group.

**Results:**

The search strategy yielded 653 papers, of which 417 were duplicates. Following analysis of the abstracts, 119 papers met the inclusion criteria. The majority of papers reported cross-sectional studies (n = 117) with three of longitudinal design. Fifteen studies which had used the original version of the measures in their original language were included in the COSMIN analysis. The most frequently used measure was the CPQ. Reliability and construct validity appear to be adequate for all three measures. Children were not fully involved in item generation which may compromise their content validity. Internal consistency was measured using classic test theory with no evidence of modern psychometric techniques being used to test unidimensionality of the measures included in the COSMIN analysis.

**Conclusion:**

The three measures evaluated appear to be able to discriminate between groups. CPQ has been most widely tested and several versions are available. COHIP employed a rigorous development strategy but has been tested in fewer populations. C-OIDP is shorter and has been used successfully in epidemiological studies. Further testing using modern psychometric techniques such as item response theory is recommended. Future developments should also focus on the development of measures which can evaluate longitudinal change.

## Background

Patient reported outcomes can be defined as: “reports coming directly from patients about how they feel or function in relation to a health condition and its therapy without interpretation by healthcare professionals or anyone else” [1]. The drive for the use of patient reported outcome measures (PROMs) has come from the shift from a biomedical perspective to a broader biopsychosocial model of health [2]. The proposed benefits of such an approach to patient care are [3]:

1. patients themselves are in the best position to assess the improvement in their symptoms or quality of life

2. involving patients in their healthcare

3. observer bias can be reduced

4. consideration of patients’ views increases public accountability

PROMs were initially developed for use in research and following this further developed by clinicians to allow evaluation of individual patients. The increasing prioritisation of this approach to patient care allows the patient’s perception of the effects of clinical intervention to be understood by both clinicians and researchers [4]. As many dental conditions have psychological and social implications, the use of such instruments in dentistry is particularly appropriate [5].

As the development of such measures has increased, several groups have produced guidelines for PROMs in an attempt to aid appraisal and appropriate selection of these instruments. The Scientific Advisory Trust of the Medical Outcomes Trust initially published a set of criteria for assessment of health status and quality of life measures in 1996 [6]. These were updated in 2002 to reflect the emerging techniques being used in the development of these measures [7]. The authors suggest eight key areas for consideration (conceptual and measurement model; reliability; validity; responsiveness; interpretability; respondent and administrative burden; alternate forms and cultural and language adaptations) and criteria against which measures can be reviewed. These guidelines were developed to help the Medical Outcomes Trust (MOT) to evaluate new measures submitted to them, to ascertain which were suitable for dissemination. However, although they provide clear information regarding areas to be assessed, no specific quality standards were included.

More recently a checklist has been produced by the Consensus-based Standards for the Selection of Health Measurement Instruments initiative (COSMIN) which allows articles reporting on the evaluation of PROMs to be evaluated against defined criteria [8]. It is hoped that the use of this checklist will standardise systematic reviews of PROMs and identify areas for refinement. The categories match those of the MOT and the group has also produced explicit quality criteria for each category [9]. These criteria are shown in Table 1.

**Table 1:** **Quality criteria based on those proposed by Terwee and colleagues **[9]

**Property**	Quality criteria*
Content validity	+ a clear description is provided of the aim of the measure, the target population, concepts being measured and involvement of the target population and/or investigators or experts in item selection
	? A clear description of the above is lacking or only target population involved or doubtful design or method
	- No target population involvement
	0 No information on target population
Internal consistency	+ Factor analyses on adequate sample size (7x the number of items and >100) and Cronbach’s alpha calculated per dimension and between 0.7 and 0.95
	? No factor analysis or doubtful design or method
	- Cronbach’s alpha <0.7 or >0.95
	0 No information found on internal consistency
Criterion validity	+ Convincing argument that there is a “gold standard” and correlation >0.7
	? No convincing argument that gold standard truly is “gold” or doubtful design or method
	- Correlation with gold standard <0.7
	0 No information on criterion validity
Construct validity	+ Specific hypotheses were formulated and at least 75% of the results are in accordance with these
	? Doubtful design or method
	- Less than 75% hypotheses confirmed
	0 No information on construct validity
Reproducibility	*Agreement*
	+ MIC > SDC or MIC outside LOA or convincing arguments that agreement is acceptable
	? Doubtful design or method or above not fulfilled
	- MIC > SDC or MIC equals or inside LOA
	0 No information found on agreement
	*Reliability*
	+ ICC or weighted Kappa >0.7
	? Doubtful design or method (e.g. time interval not mentioned)
	- ICC or weighted Kappa <0.7
	0 No information on reliability
Responsiveness	+ SDC < MIC or MIC outside LOA or RR > 1.96 or AUC > 0.7
	? Doubtful design or method
	- SDC > MIC or MIC equals or inside LOA or RR < 1.96 or AUC < 0.7
	0 No information on responsiveness
Floor or ceiling effects	+ < 15% of the respondents achieved the highest or lowest scores
	? Doubtful design or method
	- > 15% of the respondents achieved the highest or lowest scores
	0 No information found on interpretation
Interpretability	+ Mean and SD scores presented for at least four relevant subgroups of patients and MIC defined
	? Doubtful design or method or less than four subgroups or no MIC defined
	0 No information on interpretation

MIC = Minimal important change; SDC = smallest detectable change; LOA = limits of agreement; ICC = intraclass correlation; SD = standard deviation.

+ = positive rating; ? = Indeterminate rating; - = negative rating; 0 = no information available. *Doubtful design or method = lacking a clear description of the design or methods of the study, sample size smaller then 50 subjects or any other important methodological weakness in design or execution of the study.

Over the past few decades there have been many PROMs produced, which purport to measure oral health-related quality of life (OHRQoL). OHRQoL was defined by Locker and Allen [10] as “The impact of oral diseases and disorders on aspects of everyday life that a patient or person values, that are of sufficient magnitude, in terms of frequency, severity or duration to affect their experience and perception of their life overall” [10]. However, a number of the questionnaires developed have involved only limited input from lay people. Therefore they may be more accurately described as measures of oral health status, as without patient involvement in their development it is difficult to ascertain whether the items accurately reflect what is important to patients [10].

The application of measures can vary according to the aim of the investigation, for example, they may be used to influence health and social policy, assess the impact of different treatment regimens or be used to analyse change in individual patients over time (Table 2).

**Table 2:** **Summary of the applications of OHRQoL measures proposed by Robinson and co-workers **[11]

**Theoretical**	Exploring models of oral health
	Describing factors influential to health
**Political**	Demonstrating involvement of the public in healthcare
	Identifying the public’s priorities
	Advocacy
**Practical**	Planning, monitoring and evaluating services
Public health	Needs assessments
Research	Evaluating outcomes of healthcare interventions
Clinic based	Evaluating individual patient care
	Improving patient-practitioner communication
	Clinical audit
	Marketing of services

Although the criteria proposed by the MOT and the COSMIN group address the psychometric properties of outcome measures, they do not specifically focus on aspects relating to the purpose and patient-centred nature of the instruments and thus whether they contain items which may reflect OHRQoL. Locker and Allen [10] performed a review of OHRQoL measures using criteria modified from those suggested by Gill and Feinstein [12] and Guyatt and Cook [13] in order to explore these factors [10,12,13]. Specific questions were as follows:

1. Is the stated aim to measure OHRQoL and is this explicit? If so, are these constructs defined and are the constituent domains identified?

2. If not, is there an alternative construct measured by the instrument specified and defined and its constituent domains identified?

3. Do the investigators specify the contexts in which the measure is to be used? Was it developed for use with groups (as in surveys or clinical trials) or individuals (as in clinical practice)?

4. Were the items comprising the questionnaire derived from qualitative interviews with those intended to complete the questionnaire?

5. Is there evidence that the aspects of life the items address are important to those who will be completing the questionnaire?

6. Does the questionnaire contain global ratings of health-related quality of life or quality of life?

7. How was the measure validated? Was it tested against oral health indicators or were broader indicators that may capture aspects of quality of life used? Is the stated aim to measure OHRQoL and is this explicit? If so, are these constructs defined and there constituent domains identified.

The review found that, although the measures covered a variety of areas such as functional and psychosocial aspects of oral health, there was a degree of uncertainty regarding whether they actually measured OHRQoL or quality of life.

Following the development of measures for use in adults, several questionnaires have been produced for use with children or using parents as proxies. These generic questionnaires are designed to cover a variety of oral conditions such as dental caries, malocclusion and craniofacial anomalies. They include the Child Perceptions Questionnaire (CPQ) [14-16], the Child Oral Impacts on Daily Performances Index (C-OIDP) [17], the Child Oral Health Impact Profile (COHIP) [18], the Early Child Oral Health Impact Scale (ECOHIS) [19] and the Scale of Oral Health Outcomes for 5-year-old children (SOHO-5) [20], the Michigan Oral Health-Related Quality of Life scale (MOHRQoL) [21] and the Pediatric Oral Health-Related Quality of Life Measure (POQL) [22]. All but the MOHRQoL and ECOHIS are designed for self-report.

The most frequently used measures for self-completion by children are the CPQ, the C-OIDP and COHIP. These measures were chosen for inclusion in this review as they cover a wide age range and variety of conditions and therefore most likely to be of use in a range of studies. Measures which are completed by proxies were not included as it has been demonstrated that there may be discrepancies between proxy scores and those provided by children themselves [23-25]. The CPQ is part of a battery of questionnaires for children and their carers [14-16]. There are versions for 11-14-year-olds, 8-10-year-olds and four short forms based on the measure for 11-14-year-olds. The C-OIDP was adapted for use in children from the Oral Impacts on Daily Performances index which is frequently used in adult populations [17]. Finally the COHIP, is designed for 8-15-year-olds and was derived from the same initial item list as the CPQ [18].

Although these measures are frequently used and have been translated into many different languages, to date there has been no review of their development, validation and use. Therefore the aim was to assess the methodological quality of the development and testing of CPQ, C-OIDP and COHIP. To fulfil this aim, the specific objectives were to:

1. describe these measures and their use

2. assess the methodological quality and measurement properties against existing criteria.

The criteria used were based on those described by Locker and Allen and COSMIN criteria [8-10]. The findings of this study will help researchers select the most appropriate measure to use in future projects and provide recommendations for refinement of these measures.

## Methods

### Search strategy

A systematic search strategy was used to identify eligible studies, using the Mesh terms “child” and “quality of life” in combination with the names or the commonly used acronyms of the three measures. Both MEDLINE (through PubMed) and Web of Science were used to search for articles published up to December 2012. Reference lists of included studies were also searched to identify additional studies.

### Selection criteria

Titles and abstracts were read independently by two investigators (FG and ZM) to ascertain whether they met the inclusion criteria. Disagreements were resolved by discussion and where doubt existed, the full paper was retrieved. A paper was judged to be suitable for inclusion if:

• it used either the CPQ, COHIP or C-OIDP (or versions of them)

• it included participants aged 16 years or younger

• the measures were completed by the participants, not proxies

• the full paper was available in English

• it reported primary data

### Data collection

1. Description of measures and their use (Objective 1)

To fulfil objective one and describe the measures and their use, data were collected relating to:

• the aim of the study

• the measure used

• study type (for example; development, validation, cross cultural adaptation, etc.)

• population (i.e. clinical, school-based)

• measurement properties (detailed below)

• development of the measure, described using the criteria proposed by Locker and Allen [10]

Results were collected by two teams of two investigators (FG/HDR and ZM/CD) for all included studies. A protocol, with description of the data required to be collected was produced. The data collection spreadsheet was piloted using 10 articles, following which descriptors were added to each of the categories to aid completion. A training exercise was then held with all investigators to ensure consistency of data extraction. Where there was disagreement between investigators, this was resolved by discussion to reach a consensus.

2. Assessment of the methodological quality of the development and testing of measures (Objective 2)

The COSMIN checklist was used to evaluate the quality of studies that reported the development or evaluation of the original form of the CPQ, COHIP or C-OIDP in the original language [8]. This tool allows the methodological quality of studies to be assessed against criteria for each measurement property and has been used successfully in systematic reviews of outcome measures [26,27]. The checklist contains 5–18 items per property which are rated excellent, good, fair or poor, with the lowest score for any item being assigned as the overall score for that property.

Two reviewers (FG and ZM) decided which properties had been assessed in each study and assigned an overall score. A calibration exercise was held prior to data collection to ensure consistency. Disagreements were resolved by discussion between investigators to reach a consensus. Both intra- and inter-examiner reliability were assessed and were found to be excellent (weighted Kappa = >0.9).

#### Quality assessment rating

The rating system proposed by Terwee and colleagues [9] was used to assess the quality of the instruments using the results of the studies evaluated by the COSMIN checklist. This allows a positive, negative or indeterminate rating to be assigned depending on the published results (Table 1).

#### Measurement properties analysis

Validity, reliability, responsiveness and interpretability of the measures were analysed using the following aspects [9]:

• Content validity: The degree to which the items in the questionnaire are a reflection of those important to the study population and to the construct under scrutiny. Four main areas were assessed:

1. Was the measurement aim stated, for example; is the questionnaire designed to be discriminative, evaluative or predictive?

2. The concept which the questionnaire was designed to measure is stated so that others can use it appropriately.

3. Methods for item selection and reduction are justified and should include the target population.

4. Interpretability of the questions, for example, these should be age-appropriate and should not require reading skills above that of a 12-year-old where they are designed for adults.

• Construct validity: this refers to the extent to which scores relate to other measures of a similar concept under scrutiny and should be tested using predefined hypotheses to avoid bias.

• Internal consistency: the extent to which items in the questionnaire measure the same construct. In classic test theory, this is expressed using Cronbach’s alpha value. A low Cronbach’s alpha indicates a lack of correlation between items on the scale, meaning that combining them to give an overall score is not meaningful. Whereas, a very high value indicates excellent correlation, therefore some items may be redundant. Values of 0.7 to 0.95 are deemed to be acceptable for research tools. Principal component analysis or exploratory factor analysis, followed by confirmatory factor analysis are the preferred methods for attaining homogenous scales, as these allow redundant items to be removed and can be used to identify the number of subscales present. Criterion validity: this relates to whether the scores on a particular questionnaire have a positive correlation with a gold standard. There are no gold standards in the field of OHRQoL and therefore measurement of this is only appropriate when testing a short form against the existing measure.

• Test-retest reliability: the ability of the measure to produce reproducible results in a stable population over time. The time between administrations should be long enough to prevent recall but short enough to minimise changes in clinical status. One to two weeks is usually adequate, however, the clinical concern under investigation may require a different time interval, for example, in palliative care where deterioration in a patient’s health may occur rapidly. The most suitable expression of this value is using the Intraclass Correlation Coefficient (ICC). Values greater than or equal to 0.7 are deemed acceptable.

• Responsiveness: the ability of a questionnaire to detect clinically important changes over time, for example, after an intervention. Predefined hypotheses should be defined and tested.

• Floor or ceiling effects: these were considered to be present where more than 15% of patients score the highest or lowest score possible. Where this is present, there may be issues with content validity as extreme ends of the scale are not represented. In addition, participants who achieved the lowest or highest scores cannot be distinguished from each other, reducing reliability.

• Interpretability: the degree to which scores on the questionnaire can be given qualitative meaning. For example, the provision of means and standard deviation of scores of relevant subgroups (clinical diagnoses, age groups, gender).

#### Best evidence synthesis

A best evidence synthesis was performed to summarise the evidence for each measure based on the methodological quality, consistency of results and the number of studies.

Two reviewers (FG and ZM) assessed the evidence for each measure and assigned a rating. A training exercise was held to ensure consistency. Disagreements were resolved by discussion between investigators to reach a consensus.

The results were defined as:

• strong evidence: consistent findings in multiple studies of good methodological quality or one study of excellent quality

• moderate evidence: consistent findings in multiple studies of fair methodological quality or one study of good quality

• limited evidence: one study of fair methodological quality.

Where there were only studies with poor methodological quality or where statistical methods other than those recommended were used, a lack of evidence was noted.

## Results

**Figure 1:**
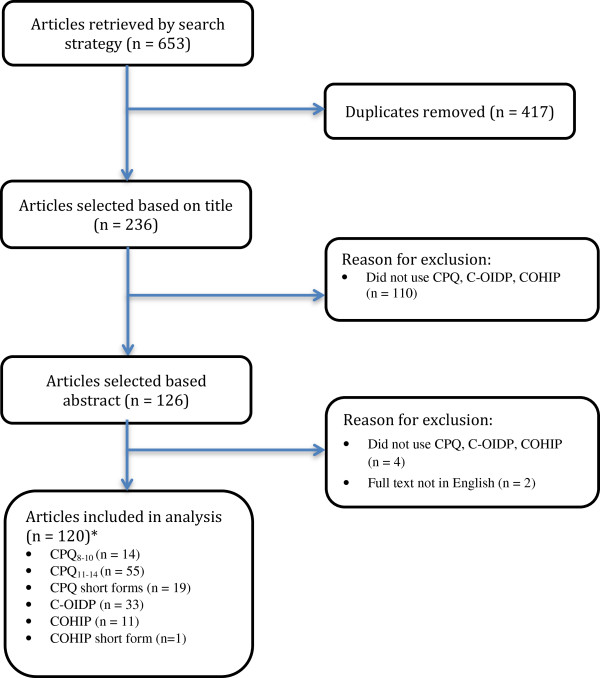
**Flowchart detailing included articles in main study.** CPQ = Child Perceptions Questionnaire. C-OIDP = Child Oral Impacts on Daily Performances Index. COHIP = Child Oral Health Impact Profile. *Some papers used more than one measure.

**Figure 2:**
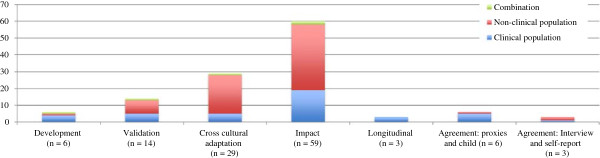
Aim of studies described by each paper and characteristics of study population (n = 120).

The search strategy yielded 653 papers. Four hundred and seventeen were duplicates leaving a total of 236 abstracts. Following analysis of the abstracts, 126 full papers which appeared to meet the inclusion criteria were retrieved. Of these, six were excluded as they did not meet the inclusion criteria therefore 120 papers were included in the analysis (Figure 1). The majority used a version of the CPQ, most frequently the original version of CPQ_11–14_ (Figure 1). Most papers reported cross-sectional studies (n = 117) with three of longitudinal design (Figure 2). The number of publications using these measures steadily increased from 2008–2011 and reached a peak of 21 in 2011. A decline, perhaps related to delays in indexing of the databases, was seen in 2012.

Fifteen studies which had used the original version of the measures in their original language were included in the COSMIN analysis. The following subsections will present findings relating to the evaluation of each questionnaire with the additional COSMIN analysis.

### CPQ [14-16,28-100]

This questionnaire was developed in Canada and was originally validated in children with caries, malocclusion and craniofacial anomalies. A number of versions have been produced. The original item pool was developed following a review of existing oral health and paediatric measures. This was further reduced following discussion with healthcare professionals, parents of children and children with a variety of oral conditions.

### CPQ_11–14_

### Description of CPQ_11–14_ and its uses

The aim of this questionnaire was to “produce a measure which conformed to contemporary concepts of child health and had discriminative and evaluative properties, and which is applicable to children with various dental, oral and oro-facial disorders”. Although not explicitly stated, the measure must therefore have been designed to measure change at a group level due to its aims. Potential items were divided into four domains: oral symptoms, functional limitations, emotional well-being and social well-being. An item impact study involving 82 children was used to reduce the number of items to 37 across the four domains. In addition, two global questions are included relating to the participant’s opinion of how their teeth and mouth affected their life overall and their perceived oral health status. The questions ask participants about the frequency of events in the previous three months and are scored on a five-point Likert scale from 0–4. A higher score indicates increased impact. The measure was validated by comparing scores between groups (caries, malocclusion, craniofacial) and by correlating overall scores with global ratings. Further details are shown in Table 3.

**Table 3:** Characteristics of included measures

**Questionnaire**	**Age range designed for in years**	**Number of items**	**Number of domains**	**Range of possible scores**	**Scoring method**	**Completion method**	**Recall period**
**CPQ**_**11–14**_	11-14	37	4	0-148	5-point Likert scale (0–4)	Self	3 months
**CPQ**_**8–10**_	8-10	25	4	0-100	5-point Likert scale (0–4)	Self	4 weeks
**CPQ**_**11–14**_**short forms**	11-14	16 or 8	4	16 item = 0-64	5-point Likert scale (0–4)	Self	3 months
				8 item = 0-32			
**C-OIDP**		8	1	0-72	4-point Likert scale (0–3)	Interview	3 months
**COHIP**	8-15	34	5	0-136	5-point Likert scale (0–4)	Self	3 months
**COHIP short form**	8-15	19	5	0-76	5-point Likert scale (0–4)	Self	3 months

CPQ = Child Perceptions Questionnaire; COHIP = Child Oral Health Impact Profile; C-OIDP = Child Oral Impacts on Daily Performances index.

#### Study types/populations

Fifty five papers used CPQ_11–14_. Of these, one described development of the measure and seven its validation. Cross-cultural adaptation and validation of these versions were described in 12 studies from Hong Kong, Brazil, Denmark, Uganda, Saudi Arabia, Thailand and Germany. One paper investigated agreement between self- and interview-administered versions, three studies analysed agreement between parent and child and one study reported on the changes in scores during orthodontic treatment. The remaining articles described OHRQoL in cross-sectional population studies and explored the impact of various dental and medical conditions.

CPQ_11–14_ had been translated into Chinese, Brazilian-Portuguese, Danish, Luganda, Arabic, Thai and German. Further versions in Malay, Finnish, Norwegian and Russian, were described but no details were provided regarding their validation.

#### Measurement properties

Twelve studies reported test-retest reliability with ICCs ranging from 0.6 to 0.94. The test-retest period varied from one week to one month and involved between 14 and 84 participants.

Internal consistency was investigated in 20 studies for CPQ_11–14_ with Cronbach’s alpha ranging from 0.72 to 0.95.

Criterion validity testing was not appropriate for this measure as there is no gold standard. Construct validity was measured using global ratings and clinical data. Positive correlations were found with global ratings but conflicting results were reported for correlations with clinical data.

No studies reported face or content validity testing, except during the development and cross-cultural adaptation of the measures.

Specific details regarding floor and ceiling effects were reported in only seven studies, with maximum proportions of 3 and 5% scoring zero or the maximum scores respectively.

Although one study reported longitudinal data, there was no reflection of what would be considered a clinically important change in score.

Mean and subgroup scores, where available, are shown in Additional file 1.

### Assessment of the methodological quality of the development and testing of CPQ_11–14_

The CPQ_11–14_ was studied in four papers in children with dental caries, enamel defects, malocclusion and craniofacial disorders. The original form has been validated in Canada and the UK.

#### Validity

Hypothesis testing for construct validity was performed in all four studies using correlations with clinical data and global ratings. The methodology was rated excellent in two cases [70,76] and fair in the other two cases [14,77]. The results for construct validity were rated positively in all studies. Content validity was considered in one study of fair methodology and rated positively [14]. Criterion validity was not applicable for this measure as there is no gold standard.

#### Reliability

Internal consistency was analysed in all four studies and the methodology rated as poor in all, as the studies did not report testing of unidimensionality by factor analysis or item response theory. Therefore internal consistency was rated as indeterminate, however, it should be noted that all studies reported Cronbach’s alpha of between 0.7 and 0.95. Test-rest reliability was performed in three studies, one of which was rated as good [70], one fair [14] and one poor [76] and all had a positive ICCs.

#### Best evidence synthesis

Combining the results of the methodological quality with the published results produced strong evidence for construct validity and lack of floor or ceiling effects, limited evidence for interpretability, reliability and content validity and a lack of evidence for internal consistency (Table 4).

**Table 4:** Best evidence synthesis per questionnaire

**Questionnaire**	**Content validity**	**Internal consistency**	**Construct validity**	**Reliability**
**CPQ**_**11–14**_	+	?	+++	+
**CPQ**_**8–10**_	?	+	++	+
**CPQ**_**11–14**_**short forms**	n/a	?	+	+
**COHIP**	+++	?	+++	+
**C-OIDP**	+	?	+	+

+++ or --- = strong evidence of positive or negative result respectively; ++ or – = moderate evidence of positive or negative result respectively; + or – = limited evidence of positive or negative result respectively; ± = conflicting results; ? = unknown due to poor methodological quality of study, n/a = no information available.

### CPQ_8–10_

### Description of CPQ_8–10_ and its uses

The aim of this measure was not explicitly stated, but it was assumed to be the same as that for CPQ_11–14_. Questions for this version were derived by a child psychologist, teachers and parents from the questions in the CPQ_11–14,_ with no input from children. This resulted in a questionnaire with 25 items across the same four domains as CPQ_11–14_. The questions are scored in the same way as CPQ_11–14_, but the response period is the last four weeks rather than the previous three months. Testing of the measure took the same approach as described above for CPQ_11–14_ (Table 3).

#### Study types/populations

Fifteen included studies had used CPQ_8–10_. One reported its development, two its validation and four were cross-cultural adaptations in Brazil, Denmark and Mexico. One was a longitudinal investigation of children following atraumatic restorative technique and one study measured agreement between self- and interview-administered questionnaires. The remainder (n = 6) described the impact of temporomandibular dysfunction, caries, fluorosis, neutropenia, malocclusion and OHRQoL of cancer survivors.

CPQ_8–10_ is available in Brazilian-Portuguese, Danish and Spanish.

#### Measurement properties

Four studies investigated test-retest reliability with ICCs ranging from 0.67 to 0.96. Retest periods ranged from seven days to two weeks with between 33 and 162 partcipants.

Eight papers reported internal consistency with Cronbach’s alpha values ranging between 0.82 and 0.95.

Construct validity was tested using correlations between global ratings, proxy measures and clinical data. All showed mainly positive correlations. Criterion validity assessment was not appropriate for this measure as there is no gold standard. No studies reported face or content validity testing.

Only one article discussed floor and ceiling effects and reported that none were found.

One study reported longitudinal data, however, no details of the magnitude of change that would be considered clinically important were discussed.

Details regarding mean and subgroup scores are shown in Additional file 1.

### Assessment of the methodological quality of the development and testing of CPQ_8–10_

Two studies reported findings from the CPQ_8–10_ in children: one involved participants with craniofacial disorders in Canada and one included a school population in Northern Ireland.

#### Validity

Hypothesis testing for construct validity was performed in both studies and was found to be positive using global ratings [15,52] and other measures of similar constructs [52]. The methodology of one study was rated good [15] and fair for the other [52]. Development of the content of the measure did not involve the target population (i.e. children) and therefore the methodology was rated poor and it was assessed as being negative for quality. Testing of criterion validity was not appropriate for this measure as there is no gold standard.

#### Reliability

Internal consistency was analysed in both studies, with factor analysis employed in one [52] which was rated fair for methodology and therefore rated positively for the measurement property. The other [15] was rated poor as there was no analysis of unidimensionality. Both studies had acceptable Cronbach’s alpha values. One study tested test-retest reliability which was rated fair methodologically and given a positive rating for reliability.

#### Best evidence synthesis

Combining the results of methodological quality with the published results demonstrated there was moderate evidence of positive construct validity, limited positive evidence to support internal consistency, reliability, interpretability and lack of floor/ceiling effects and no evidence to support adequate content validity (Table 4).

### CPQ short forms

### Description of CPQ short forms and their uses

Four short forms are available, two with 16 items and two with eight items, each derived from the questionnaires for 11-14-year-olds. An eight and 16-item version were produced using item impact data from the original study resulting in questionnaires containing two and four items per domain respectively. These are termed the “impact short forms” (ISF:8 or ISF:16). The other versions were developed using the original validation data and by selecting the two or four items contributing most to the coefficient of variation for each domain, and called the “regression short forms” (RSF:8 or RSF:16). All short forms are scored in the same way as the original version with a recall period of three months (Table 3).

#### Study types/populations

Nineteen studies were identified where the CPQ short forms or other abbreviated versions had been used. One described development, two validation and three were cross-cultural adaptions from Hong Kong, Brazil and Brunei. The remainder reported the impact of dental trauma, orthodontic treatment and socioeconomic disparities in OHRQoL.

The short forms of CPQ_11–14_ have been translated into Chinese, Brazilian-Portuguese and Malay.

#### Measurement properties

Three studies investigated test-retest reliability with ICCs ranging from 0.5 to 0.98. All reported periods between tests were two weeks and involved either 34 or 86 participants (one study did not report the number of participants).

Internal consistency was reported in six studies with Cronbach’s alpha values ranging from 0.5 (RSF:8) to 0.9 (ISF:16).

One study investigated face and content validity of the ISF:16 in an orthodontic population in the UK. This enquiry found a number of the items to be irrelevant especially with regard to the domains of oral symptoms and functional limitations. The participants also felt there were a number of items of importance to them which had been omitted.

Criterion validity was examined against the full version and found to be positive. Construct validity was assessed using global ratings and clinical data. Positive correlations with global rating were consistently found, however, there were conflicting data for correlations with clinical status. Mean and subgroup scores are shown in Additional file 1.

### Assessment of the methodological quality of the development and testing of CPQ short forms

Two studies investigated all short forms of the CPQ_11–14,_ one in a clinical population in Canada including children with caries, malocclusion and craniofacial disorders and the other in a school in New Zealand. The third study investigated face content validity of the ISF:16 in children undergoing orthodontic treatment in the UK.

#### Validity

Hypothesis testing for construct validity was undertaken in two studies using clinical data and global ratings [16,48] both of which had fair methodology and were rated positively. Criterion validity was tested in both studies against the original measure and was found to be positive with a fair methodology in both studies. The investigation of content validity [69] had excellent methodology and found that some items were irrelevant to the target population and therefore this was rated negative.

#### Reliability

Both studies [16,48] which investigated internal consistency were rated poor for methodology and were subsequently given an indeterminate rating for the measurement property. Only one study [16] analysed test-retest reliability which was given a positive rating and graded as having fair methodology.

#### Best evidence synthesis

Combining the elements from the methological quality rating and the published results, moderate evidence was found to support the lack of floor/ceiling effects, limited positive evidence for construct validity, reliability and interpretability. There was no evidence to support adequate internal consistency. There was strong evidence that content validity was inadequate in an orthodontic population for the ISF:16 version in the UK (Table 4).

### C-OIDP [17,40,101-131]

#### Description of C-OIDP and its uses

This measure was developed from the existing Thai version of the Oral Impacts on Daily Performances index (OIDP). It can be assumed that the aim of C-OIDP was to be a socio-dental health indicator (measuring the social effect of dental conditions) based on the theoretical model of oral health consequences, like the OIDP it was based on [132]. Modifications were made following face and content validity testing with Thai school children [17]. This resulted in a version with eight items with pictures to aid comprehension. The recall period was shortened from six to three months and scored on a three-point Likert scale. Participants are asked to rate both the severity and the frequency of their oral impact. The two scores can then be combined to give an overall score. Validity was tested using correlations with two global questions (perceived oral health problems and perceived treatment need). Further details are shown in Table 3.

##### Study type/populations

Thirty-three papers reported use of C-OIDP. One reported development and two its subsequent evaluation. Ten articles described cross cultural adaptation and subsequent validation in the United Kingdom, Malaysia, France, the Sudan, Tanzania, Spain, Italy, Brazil and Peru. Two studies investigated the level of agreement between self- and interview-administrations and one gave an account of changes in impact following treatment of caries. The remainder described the impact of various oral and medical conditions.

C-OIDP is available in English, French, Malay, Arabic, Kiswahili, Spanish, Portuguese and Italian. A further paper described its use in Hungary, however, no details were given regarding the validation of this version.

##### Measurement properties

Eighteen studies reported test-retest reliability with ICCs ranging from 0.7 to 0.98. Test intervals ranged from the same day to three weeks with between 18 and 106 participants.

Five studies reported internal consistency for C-OIDP with Cronbach’s alpha values ranging between 0.79 and 0.91.

Construct validity was tested using perception of treatment need, satisfaction with oral health, dental appearance and clinical data. Significant correlations were found with perceived need for treatment, oral hygiene and satisfaction with oral health. Testing of criterion validity was not appropriate for this measure. There were no studies which reported investigation of face or content validity.

There were no studies which reported the incidence of floor or ceiling effects.

One study was longitudinal in design, however, no data were available regarding changes in score which were considered clinically significant.

Additional file 2 provides mean and subgroup scores, where available.

### Assessment of the methodological quality of the development and testing of C-OIDP

This questionnaire had been analysed in two studies involving Thai school children and were evaluated using the COSMIN criteria.

#### Validity

Development of the content of the measure was rated as fair and given a positive rating [17]. Hypothesis testing for construct validity was undertaken in both studies using clinical data [102] and perceived oral health need [17,102] with good methodology and positive findings. Criterion validity testing was not appropriate for this measure as there is no gold standard.

#### Reliability

Testing of internal consistency and test-retest reliability were tested in one study [17]. Assessment of internal consistency was rated poor methodologically due to lack of testing for unidimensionality and therefore given an indeterminate rating, although Cronbach’s alpha was adequate. Reliability testing was rated fair for methodological quality and given a positive rating for the published results.

#### Best evidence synthesis

Limited positive evidence was available to support construct validity, there was limited evidence of positive reliability and interpretability and no evidence for internal consistency (Table 4).

### COHIP [18,25,132-140]

#### Description of COHIP and its uses

This instrument was designed for use in clinical situations to discriminate between children with different clinical conditions and with differing clinical severity. It was intended that it should be used in research and in clinical practice. The questionnaire was produced using the same initial item pool as CPQ. It was developed in Canada and in the US, with versions in English, French and Spanish. Item reduction was carried out in four phases with healthcare professionals, children and caregivers participating in interviews and item impact studies. Following this factor analysis was performed to finalise the items. The final questionnaire contained 34 items across five domains (oral health, functional well-being, social–emotional well-being, school environment and self-image). Participants are asked to report on the frequency of events over the past three months on a five-point Likert scale which is scored from 0–4. There are both positive and negative items, with negative items having their scoring reversed, therefore lower scores reflect worse OHRQoL. Validity was tested using comparisons between groups (caries, malocclusion and craniofacial), and those with differing levels of clinical severity. Correlation with other instruments and the two global rating questions (treatment expectations and effect on life overall) were also undertaken. Further details are available in Table 3.

##### Study types/populations

Eleven articles included COHIP. One study reported development of the measure and two its validation. Four described cross-cultural validation in Korea, Iran and the Netherlands. The remaining studies described investigation of the impact of orthodontic treatment, correlations with self-reported dental aesthetics, the impact of cleft lip and palate and concordance between child and caregiver’s scores.

COHIP has been translated into Dutch, Korean, Malay and Farsi. Finnish, Norwegian and Russian versions have also been reported but the lacked validation data for these translated measures.

##### Measurement properties

Two studies assessed test-retest reliability with ICCs ranging from 0.84 to 0.88, one using a two-week and the other a three-week interval between tests. The number of patients involved was not defined in either study.

Construct validity was measured in four studies, using correlations with global ratings of general and oral health, clinical data and parental scores. Statistically significant correlations were found between global ratings, number of decayed surfaces and degree of overjet.

As there is no gold standard, testing of criterion validity was not appropriate for this measure. No data were available for face or content validity outwith the initial development stage.

Two studies reported the proportion of floor (0–0.4%) and ceiling effects (0%).

There were no longitudinal studies and therefore there are no responsiveness data available for this measure.

Mean and subgroup scores are shown in Additional file 3.

### Assessment of the methodological quality of the development and testing of COHIP

Three studies investigated the COHIP in clinical and school populations in Canada and USA.

#### Validity

The methodology for development of the content of the questionnaire was rated as excellent and found to be positive [18]. Hypothesis testing for construct validity was investigated in two studies [135,137] with excellent methodology and was positive.

#### Reliability

One study [135], investigated internal consistency, this had a good methodology but did not test unidimensionality in this population and was therefore rated indeterminate. The same study [135] investigated test-retest reliability and was rated fair with a positive result.

#### Best evidence synthesis

There was strong positive evidence of adequate content validity and construct validity and limited positive evidence of reliability, interpretability and lack of floor/ceiling effects. Although factor analysis had been performed during the development of the measure, to aid item reduction, further investigation of the unidimensionality of the scale had not been performed and therefore internal consistency was rated as indeterminate (Table 4).

### COHIP short form [141]

#### Description of COHIP short form and its uses

Recently a 19 item short form of the COHIP has been developed by using confirmatory factor analysis to remove items with weak loadings. This version had not been tested independently at the time of this review.

#### Assessment of the methodological quality of the development and testing of COHIP short form

This measure has been evaluated only in the study used to develop the measure [141]. Data from the original version were used to evaluate the measure.

##### Validity

Hypothesis testing for construct validity using clinical data, parental and global ratings revealed positive results with a fair methodology. Criterion validity was not assessed despite the fact that the original form would be considered the gold standard.

##### Reliability

Confirmatory factor analysis was used to determine the items for inclusion in the short form, therefore the measure was given a positive rating for internal consistency with a fair methodology.

##### Best evidence synthesis

This was not evaluated due to the limited evidence for this measure at the time of this review.

## Discussion

This review evaluated the three most commonly used generic measures of OHRQoL for children against existing criteria. The CPQ_11–14_ was found to be the most frequently employed measure. In the main, questionnaire use has been restricted to validation, cross cultural adaptation and the description of impacts in various conditions. Thus, many of their potential applications, such as those described in Table 2, have not yet been pursued. For example, the theoretical models which the questionnaires are based on have rarely been evaluated. Exploration of this facet may improve our understanding of what these questionnaires really measure [31]. In addition, few studies have explored changes following treatment and those that did, offered no information regarding clinically meaningful changes to the patients involved. Finally, their influence on policy has yet to be seen. It has been suggested that difficulty in interpretation, due to uncritical reporting of scores, has contributed to their lack of use by policy makers [142].

Although the aim of the measures seemed implicit from the outset, OHRQoL was not defined in any of the papers describing their development. As there is great debate about whether questionnaires of this type can really capture aspects of quality of life, it is important to define exactly what it is that will be measured [10]. Some authorities have suggested that measures such as these may be more appropriately termed “subjective health status measures” [10]. The incorporation of global quality of life and OHRQoL may allow patients to express their own feelings towards these concepts [143]. Analysis of this information, together with the numerical scores for the measure, may provide a way to ascertain the meaning of the scores derived from these instruments [10].

In addition, further qualitative investigation may be required to ensure that questionnaires cover the full range of issues which are important to children. Individuals with the relevant conditions should be involved in item generation [13]. Although children were involved in the development of some of these questionnaires, they did not fully participate in item generation and therefore impacts which are important to children may have been omitted. Indeed, Marshman and colleagues found that orthodontic patients felt some of the questions in the CPQ_11–14_-ISF:16 to be irrelevant or difficult to understand [69]. Participants also commented that a frequency based response format was less relevant than one which was based on severity. This was the only study to investigate these aspects outwith the development process and therefore it is not possible to generalise these findings, however, further investigation of face and content validity may be useful in other settings. It should be noted that other investigators working with children, have implemented severity based response formats following children’s involvement [144,145]. As both CPQ and COHIP rely mainly on frequency scores, this may impair their ability to adequately reflect children’s views.

It has been suggested that quality of life measures should include both “positive” and “negative” items to encompass all aspects which may impact upon well-being [146]. Indeed, it has been suggested that the inclusion of positive items may aid identification of factors relating to coping or resilience which might otherwise be difficult to ascertain [147]. Of the measures included reviewed in this paper, only COHIP incorporates positive items. These statements include “I am happy with my teeth” and “Felt that you were attractive”. Both items were suggested during focus groups with parents and further endorsed by children.

Three studies investigated change following an intervention and reported changed mean scores [80,97,118]. Discussion of whether these changes were clinically meaningful was not included. In order to evaluate responsiveness it is essential to calculate the minimal important change or difference (MID). This can be done by comparing global ratings to assess when patients perceive change to have occurred and their overall questionnaire score. Thus the MID can be defined as “the smallest difference in score, that a person perceives as important [148]. Therefore although CPQ_11–14_, CPQ_8–10_ and C-OIDP have been used longitudinally they have not been validated for use in this way. Disease specific measures have been found to be more adept at detecting these clinically important changes as the questions specifically address issues associated with one disease [149]. As CPQ, C-OIDP and COHIP are generic, they may be unable to identify subtle changes following interventions.

Methodological quality was assessed for 15 studies, most of which involved CPQ_11–14_. The majority of studies were rated as excellent, good or fair in relation to assessment of test-retest reliability, hypothesis testing for construct validity and content validity. However, lack of testing of internal consistency using factor analysis or item response theory (IRT) meant most studies were rated poor for this property. Factor analysis and item response theory allow redundant items to be removed, thus shortening the questionnaire and removing duplication, which would select more sensitive instruments and reduce participant burden. It should be noted that such techniques have been employed in studies using versions of the measures which have been subject to cross-cultural validation [31,63,90,96,138]. However, these methods have not been consistently applied to the original forms which were included in this analysis.

Best evidence synthesis shows strong positive evidence for hypothesis testing for construct validity for both CPQ_11–14_ and COHIP, indicating that they measure appropriately according to the construct they intend to measure. However, in this part of the analysis, COHIP had only been evaluated in two studies, both during its initial validation, and not in other populations.

Positive evidence for test-retest reliability was found for all measures indicating that they are reliable in stable populations. Strong evidence of content validity was best for COHIP, due to the rigorous process implemented in its development. Although as previously discussed, this could have been improved by involvement of children in the initial item generation, rather than at the item impact stage.

The measures evaluated in this review were developed before the publication of standards such as the COSMIN checklist. Therefore some elements, such as analysis using item response theory, were not included in many studies and affected their overall ratings.

### Recommendations

#### Which questionnaire?

All three measures appeared to respond appropriately when used discriminatively especially with regard to reliability and construct validity. However, based on the criteria used in this review, they all have shortcomings. It is therefore difficult to recommend one over another. However, the following may help in choosing which is right for different purposes:

• CPQ has been most widely used and therefore has the most evidence of its reliability and validity. However, due to inadequate reporting it is unclear how the scores can be generalised or their clinical significance. Inclusion of clinical data relating to the population under scrutiny, mean and subgroup scores and floor or ceiling effects is recommended in future studies to aid interpretability. Short forms are available, however, there are varying results with these four versions as to their reliability and validity.

• The COHIP was the last to be reported and has employed a rigorous development strategy. There was extensive involvement of children and redundant questions were removed by factor analysis. It has been tested the least but results are promising. However, it contains 37 questions which may constitute significant participant burden. The 19-item version may reduce this but further testing in different populations is required.

• C-OIDP is short (8 items) and would be of use in epidemiological surveys where it has been successfully used to assess oral impacts.

#### Future developments

• To further develop the field of OHRQoL, studies of interventions are required rather than cross-sectional descriptive studies. Development of an evaluative measure would be required to fulfil this objective. For example, a measure specific to the impacts of dental caries that could be used in clinical trials assessing the effectiveness of approaches to caries management.

• Any new questionnaires should be developed using the COSMIN criteria to ensure consistency in development, validation and reporting of results.

## Conclusion

The three measures evaluated appear to have adequate reliability and validity. However, further testing using modern psychometric techniques, which have previously been applied to some translated versions, may allow them to be refined further. These generic instruments appear to be able to discriminate between groups and therefore there does not seem to be a requirement to develop further measures of this type. There remains doubt about their ability to detect change longitudinally and future efforts should focus on this property.

## Competing interests

The authors declare that they have no competing interests.

## Authors’ contributions

FG participated in the design of the study, collected and analysed data and prepared the first draft of the paper. HDR participated in the design of the study, collected data and helped with drafting the manuscript. CD participated in the design of the study, collected data and helped draft the manuscript. ZM conceived the idea, participated in the design of the study, analysed data and helped draft the manuscript. All authors have read and approved the final manuscript.

## Pre-publication history

The pre-publication history for this paper can be accessed here:

http://www.biomedcentral.com/1472-6831/14/40/prepub

## Supplementary Material

Additional file 1 Studies which used a version of the Child Perceptions Questionnaire with details of version, setting and range and mean scores. Click here for file

Additional file 2 Studies which used a version of the Child Oral Impacts on Daily Performances index with details of version, setting and range and mean scores. Click here for file

Additional file 3 Studies which used a version of the Child Oral Health Impact Profile with details of version, setting and range and mean scores. Click here for file
