# Supplementary material for: Assessment of the quality of measures of child oral health-related quality of life
Source: BMC Oral Health. 2014 Apr 23;14:40. doi: 10.1186/1472-6831-14-40 (PMC4021173; doi:10.1186/1472-6831-14-40)
Supplement: Additional file 1 — Studies which used a version of the Child Perceptions Questionnaire with details of version, setting and range and mean scores. [file 1472-6831-14-40-S1.docx]

**Additional file 1. Studies which used a version of the Child Perceptions Questionnaire with details of version, setting and range and mean scores.**

| **Author** | **Year** | **Measure used and version** | **Number of items analysed** | **Language of version** | **Study country** | **Study population** | **Item/score distribution** | **Total mean score** | **Subgroups mean score/ proportion with impacts** |
| --- | --- | --- | --- | --- | --- | --- | --- | --- | --- |
| Agou[1] | 2008 | CPQ_11-14_ |  | English | Canada | Clinical | 3-73 |  | Low socioeconomic status = 28.4  High socioeconomic status = 19.4 |
| Agou[2] | 2011 | CPQ_11-14_ |  | English | Canada | Clinical | 0-80 |  | Orthodontic treatment group at baseline = 21.05,  At follow up = 16.16  Orthodontic waiting list group baseline = 24.07  At follow-up = 23.14 |
| Aguilar-Diaz[3] | 2011 | CPQ_8-10_ | 25 | Spanish | Mexico | School |  | 16.42 | Low malocclusion = 6.39  Moderate malocclusion = 10.93  Severe = 21.03 |
| Baker[4] | 2010 | CPQ_11-14_ | 37 | Malay | Malaysia | School |  |  |  |
| Barbosa[5] | 2009 | CPQ _8-10_  CPQ_11-14_ | 25  37 | Portuguese | Brazil | School |  |  |  |
| Barbosa[6] | 2011 | CPQ_8-10_  CPQ_11-14_ | 25  37 | Portuguese | Brazil | School |  |  | CPQ_8-10_ TMD group = 20.6  Control = 13.5,  CPQ_11-14_ TMD group = 27.6  Control = 16.3 |
| Bekes[7] | 2011 | CPQ_11-14_ | 35 | German | Germany | Home survey |  | 12.6 |  |
| Bendo[8] | 2010 | CPQ_11-14_-ISF:16 | 16 | Portuguese | Brazil | School |  |  |  |
| Benson[9] | 2008 | CPQ_11-14_ | 31 | English | UK | Clinical |  | 20.4 |  |
| Brown[10] | 2006 | CPQ_11-14_ | 36 | Arabic | Saudi Arabia | Clinic | 0-102 | 24.22 | No malocclusion = 17.38  Moderate/severe malocclusion = 25.38. |
| Cheretakis[11] | 2007 | CPQ _8-10_  CPQ_11-14_ | 25 | English | Canada | Clinic | 0-58 |  | Neutropenia = 14.2  Control = 2.5 |
| Costa[12] | 2011 | CPQ_11-14_ -ISF:16 |  | Portuguese | Brazil | School | 0-42 |  | Fixed appliances = 9.5  No active treatment = 11.5 |
| de Oliveira[13] | 2008 | CPQ _11-14_  C-OIDP | 8 | English | UK | Clinic |  | CPQ = 18.41 |  |
| Do[14] | 2007 | CPQ_8-10_  CPQ_11-14_ |  | English | Australia | School |  |  | CPQ_8-10_:  dmfs/DMFS 0 =10.8  dmfs/DMFS 1-2 =9.2  dmfs/DMFS 3-4 =12.4  dmfs/DMFS 5+=10.2  Fluorosis 0=10.4  Fluorosis 1 =10.7  Fluorosis 2=10.1  Fluorosis 3=15.7  DAI ≤34=9.6  DAI ≥35=12.6  CPQ_11-14_:  dmfs/DMFS 0 =11.8  dmfs/DMFS1-2=14.5  dmfs/DMFS 3-4=15.4  dmfs/DMFS 5+=14  Fluorosis 0=15.1  Fluorosis 1=9.2  Fluorosis 2=6.4  Fluorosis 3=12.3  DAI ,34=13  DAI 35+=13.8 |
| Do[15] | 2008 | CPQ_8-10_  CPQ_11-14_ | 25  31 | English | Australia | Clinic | CPQ _8-10_ = 0-60  CPQ _11-14_ = 0-77 | CPQ8-10 = 10.7  11-14 = 13.3 |  |
| Fakhruddin[16] | 2008 | CPQ_11-14_ | 10 | English | Canada | School |  |  |  |
| Fergus[17] | 2010 | CPQ (version not stated) |  |  | Montserrat | School |  | 24 | Males = 24.28  Females = 23.44 |
| Ferreira [18] | 2012 | CPQ (abbreviated version) | 14 | Portuguese | Brazil | School | 0-37 | 11.32 |  |
| Foster-Page[19] | 2005 | CPQ_11-14_ | 35 | English | New Zealand | School | 0-103 | 17.3 | Male = 15.8  Female = 18.9  DMFS 0 = 14.4  DMFS 1 = 16.9  DMFS 2/3 = 16  DMFS ≥4 = 21.8  Malocclusion:  Minor/none = 14.8  Definite = 17.2  Severe = 19.6  Handicapping = 21.6 |
| Foster-Page[20] | 2008 | CPQ_11-14_ CPQ_11-14_-ISF/RSF:16  CPQ_11-14-_ISF/RSF:8 | 37  16  8 | English | New Zealand | School | CPQ_11-14_ = 0-110  RSF:16 = 0-52  RSF:8 = 0-23 ISF:16 = 0-47  ISF:8 = 0-24 | CPQ_11-14_ = 18.1 RSF:16 = 9.6  RSF:8 = 4.2  ISF:16 = 9.6  ISF:8 = 4.9 |  |
| Foster-Page[21] | 2011 | CPQ_11-14_ –ISF:16 | 16 | English, Malay, Portuguese | New Zealand, Brunei, Brazil | School | 0-49 |  | Northland = 11.5  Brunei = 16.8  Brazil = 12.4  Otago = 14.6 |
| Foster-Page[22] | 2012 | CPQ_11-14_- ISF:16 | 37 | English | New Zealand | School |  | 14.4 | Male = 13.2  Female = 15.7  Maori = 11.1  Non-Maori = 14.7  DMFS 0 = 13.3  DMFS 2 = 15.4  DMFS ≥3 = 16  Malocclusion:  minor/none = 13.5  definite = 14  severe = 14.3  handicapping = 15.6 |
| Foster-Page[23] | 2012 | CPQ_11-14_ |  | English | New Zealand | School |  | At 13 years = 17.1  At 16 years = 17 | At 13 years:  DMFS 0 = 15.2  DMFS 1,2,3 = 17.1  DMFS ≥4+ = 17.9  At 16 years:  DMFS 0 = 15.7  DMFS 1,2,3 = 16.9  DMFS 4+ = 19.1 |
| Goursand[24] | 2008 | CPQ_11-14_ | 37 | Portuguese | Brazil | Clinic | 0-88 | 16.23 | Without untreated = 12.89 With untreated = 24.50 |
| Gururatana [25] | 2011 | CPQ _11-14_ CPQ_11-14-_ISF/RSF:16  CPQ_11-14_-ISF/RSF:8 | 37  16  8 | Thai | Thailand | School | CPQ_11-14_ = 1-61  ISF:16 = 1-29  RSF:16 = 1-31  ISF:8 = 1-14  RSF:8 = 0-15 | CPQ_11-14_ = 24.3 ISF:16 = 13.4 RSF:16 = 14.1  ISF:8 = 6.8  RSF:8=6.9 |  |
| Humphris[26] | 2005 | CPQ _8-10_ | 25 | English | UK | School |  |  |  |
| Jokovic[27] | 2002 | CPQ _11-14_ | 37 | English | Canada | Clinic | 1-80 | 26.3 | Paediatric group = 23.3  Orthodontic group = 24.3  Orofacial group = 31.4 |
| Jokovic[28] | 2003 | CPQ _11-14_ | 31 | English | Canada | Clinic | 2-56 | 26.2 |  |
| Jokovic[29] | 2004 | CPQ _8-10_ | 25 | English | Canada | Clinic | 1-55 | 18.6 | Paediatric group = 18.4  Orofacial group = 19.1 |
| Jokovic[30] | 2005 | CPQ _11-14_ | 37 | English | Canada | Clinic |  |  |  |
| Jokovic[31] | 2006 | CPQ_11-14_  CPQ_11-14_- ISF:16  CPQ_11-14_-ISF:8 | 37  16  8 | English | Canada | Clinic | ISF:16 = 1-40  RSF:16 = 1-37  ISF:8 = 0-24 RSF:8 = 0-22 | ISF:16 = 13.8 RSF:16 = 13.6  ISF:8 = 7.4  RSF:8 = 6.6 | Paediatric group:  ISF:16 = 11.9  RSF:16 = 11.9  ISF:8 = 6.8  RSF:8 = 5.3  Orthodontic group:  ISF:16 = 13  RSF:16 = 13  ISF:8 = 7.7  RSF:8 = 6.4  Orofacial group:  ISF:16 = 16.5  RSF:16 =15.9  ISF:8 = 8.8  RSF:8 = 8 |
| Kadkhoda[32] | 2011 | CPQ_11-14_-RSF:8 | 8 | Farsi | Iran | Clinic/school | 0-22 |  | Functional appliance = 6.9  Headgear = 6.5  No malocclusion = 2.2 |
| Kohli[33] | 2011 | CPQ_11-14_ | 29 | English | USA | Clinical | 2-80 | 31.6 | Males =27  Females = 39.4 |
| Kok[34] | 2004 | CPQ_11-14_ | 37 | English | UK | School |  |  |  |
| Kolawole[35] | 2011 | CPQ_11-14_ | 37 | English | Nigeria | School | 0-81 | 23.44 | Male = 22.7,  Female = 24.17  DMFT 0 = 23.55  DMFT 1 = 18.57  DMFT 2 = 38  Dental Aesthetic Index:  Minor = 25.11  Definite = 20.19  Severe = 20.47  Handicapping = 25.77 |
| Koposova[36] | 2010 | CPQ (assume 11-14) |  | Norwegian/Russian | Norway/Russia | School |  |  | Russia = 20.2  Norway = 14.2  DMFT 0 = 14.8  DMFT >0 = 20.2 |
| Koposova[37] | 2012 | CPQ_11-14_ | 37 | Russian/Norwegian | Russia/Norway | School | Norway = 0-34  Russia = 0-85 | Norway = 9.9  Russia = 19.5 | DMFT 0 = 15.8,  DMFT >0 = 18.6 |
| Kumar[38] | 2011 | CPQ (assume 11-14) | 31 (scored 1-5) |  | India | School/orphanage |  |  | With parents = 132.92 |
| Laing[39] | 2010 | CPQ _11-14_ | 37 | English | UK | Clinic | 2-80 |  | Hypodontia = 26.82  Control = 28.52 |
| Lau[40] | 2009 | CPQ_11-14_  CPQ_11-14_- ISF/RSF:16  CPQ_11-14_-ISF/RSF:8 | 37  16  8 | Chinese | Hong Kong | School |  |  |  |
| Locker[41] | 2005 | CPQ_11-14_ | 37 | English | Canada | Clinic | 1-80 | 27.8 | Orofacial group = 31.4  Dental = 23.2 |
| Locker[42] | 2007 | CPQ _11-14_  CPQ_11-14_-ISF:16  CPQ_11-14_-ISF:8 | 35  16  8 | English | Canada | Clinic | CPQ_11-14_ = 1-75  ISF:16 = 1-41  ISF:8 = 0-19 | CPQ_11-14_ = 21.1  ISF:16 = 8.9  ISF:8 = 8.9 | Malocclusion (CPQ_11-14)_:  Minor/none = 16.1  Definite = 19.6  Severe = 17.7  Handicapping = 24.5 |
| Locker[43] | 2007 | CPQ _11-14_ | 10 | English | Canada | School | 10-32 | 12.9 | Number of carious teeth:  0 = 12.8  1 = 12.9  ≥ 2 = 14.6  Aesthestic component IOTN:  1-4 = 12.7  5-7 = 13.1  8-10 = 13.8  Fluorosis:  None = 12.7  Very mild = 13.5  Mild = 11.9 |
| Locker[44] | 2010 | CPQ_11-14_ | 37 | English | Canada | Clinic | 4-69 | 22.3 |  |
| Luoto[45] | 2009 | CPQ_11-14_ | 37 | Finnish | Finland | Clinic/school |  |  | Attending dentist:  Fear = 35.4  No fear = 25  General fear:  Fear = 25.3  No fear = 29.9  Fear of treatment:  Fear = 33.2  No fear = 24 |
| Marshman[46] | 2005 | CPQ_11-14_ | 37 | English | UK | Clinic | 3-53 | 18.07 |  |
| Marshman[47] | 2010 | CPQ_11-14_-ISF:16 | 16 | English | UK | Clinic |  |  |  |
| Martins[48] | 2009 | CPQ_8-10_ | 25 | Portuguese | Brazil | Clinic |  |  | Caries = 14.77  Malocclusion = 10.7  Both = 21.29 |
| Martins-Junior[49] | 2012 | CPQ _8-10_ | 29 | Portuguese | Brazil | School |  |  |  |
| Martins-Junior[50] | 2012 | CPQ_8-10_ | 25 | Portuguese | Brazil | School | Caries = 0-54  No caries = 2-28 |  | Caries = 23.1  No caries = 12 |
| Massarente[51] | 2008 | CPQ _11-14_ | 37 | Portuguese | Brazil | Clinic | 2-90 | 28.5 |  |
| McGrath[52] | 2008 | CPQ_11-14_ | 37 | Chinese | Hong Kong | Clinic | 7-67 | 23.1 | Paediatric group = 19.3  Orthodontic group = 26.9 |
| O'Brien[53] | 2006 | CPQ_11-14_ | 36 | English | UK | School |  |  | Medians:  Male = 50,  Female = 54  Townsend deprivation score:  Low = 52  High =52  Child’s Aesthetic component of IOTN score:  1-3 = 51  4-10=54  Dentist’s aesthetic component score of IOTN:  1-5 = 51  6-10 = 53  Dental health component of IOTN:  2-3 = 51  4-5 = 54  No orthodontic treatment = 51  Previous orthodontic treatment = 60  Current orthodontic treatment = 58  On waiting list = 50 |
| O'Brien[54] | 2007 | CPQ_11-14_ |  | English | UK | Clinic | 0-57 | Malocclusion group = 17.6 (median) Control group = 14 (median) | Crowding = 17.1 (median)  Overjet = 20 (median)  Hypodontia = 18 (median) |
| Paula[55] | 2012 | CPQ_11-14_ | 35 | Portuguese | Brazil | School | 0-106 | 23.24 |  |
| Paula[56] | 2012 | CPQ_8-10_ |  | Portuguese | Brazil | School | Controls = 0-51  Caries = 3-96  Post treatment = 0-100 |  | Controls = 19  Caries = 42  Post treatment = 29 |
| Piovesan[57] | 2010 | CPQ_11-14_ | 37 | Portuguese | Brazil | School | 0-99 | 20.9 |  |
| Piovesan[58] | 2011 | CPQ_11-14_ | 37 | Portuguese | Brazil | School |  |  |  |
| Piovesan[59] | 2012 | CPQ_11-14_ | 37 | Portuguese | Brazil | School |  | 18.4 | Trauma = 21.03,  No trauma = 20.85 |
| Porritt[60] | 2011 | CPQ_11-14_-ISF:16 | 16 | English | UK | Clinic | 0-51 | 15.5 at baseline  12.1 at follow up |  |
| Ramos-Jorge[61] | 2012 | CPQ_8-10,_  CPQ_11-14_-ISF:8 | 25  8 | Portuguese | Brazil | School |  |  | Interview 1^st^:  CPQ_8-10_ = 13.5  ISF:8= 8.7  Interview 2^nd^:  CPQ_8-10_ = 21.9  ISF:8 = 8.9  Self completion 1^st^:  CPQ_8-10_ = 12.4  ISF:8 = 8.2  Self completion 2^nd^  CPQ_8-10_ = 15.8  ISF:8 = 7.9 |
| Robinson[62] | 2005 | CPQ_11-14_ | 37 | Luganda | Uganda | School |  |  | Fluorosis score:  >2 = 33.1,  0-2 = 25.4  DMFT 0 = 21.9,  DMFT >0 = 32.5 |
| Taylor[63] | 2009 | CPQ_11-14_ | 37 | English | USA | Clinic |  |  |  |
| Torres[64] | 2009 | CPQ_11-14_-ISF:16  CPQ_11-14_-ISF:8 | 16  8 | Portuguese | Brazil | School | ISF:8 = 0-18 ISF:16 = 0-38 | ISF:8 = 6.8  ISF:16 = 11.9 |  |
| Traebert[65] | 2010 | CPQ_11-14_ | 37 | Portuguese and English | Brazil and New Zealand | School |  |  | New Zealand = 17.8  Portuguese = 28.1 |
| Traebert[66] | 2010 | CPQ_11-14_ | 37 | English | New Zealand | School | 0-110 | 18.1 | Maori = 19.1  Non-Maori = 17.9 |
| Traebert[67] | 2012 | CPQ_11-14_ short form | 16 | English | New Zealand | School | 0-49 | 12.4 | Trauma =14.6  Without = 9.6 |
| Vargas-Ferreira[68] | 2010 | CPQ _11-14_ | 37 | Portuguese | Brazil | School | 0-94 | 18.59 | Male = 17.7  Female = 19.25  White = 18.19,  Non-white = 20.32  11-12 years = 18.91  13-14 years = 17.94  No erosion = 18.5,  Erosion = 19.9 |
| Wogelius[69] | 2009 | CPQ_8-10_  CPQ_11-14_ | 25  37 | Danish | Denmark | School, clinic |  |  | CPQ_8-10_:  Healthy = 8.5  Cleft lip and palate = 7.9  Rare oral diseases =16.3  CPQ_11-14_:  Healthy = 10.5  Cleft lip and palate = 17.8  Rare oral diseases = 17.8  Fixed appliances = 24.4 |
| Wogelius[70] | 2011 | CPQ_8-10_  CPQ_11-14_ | 25  37 | Danish | Denmark | School, clinic | CPQ_8-10_ = 0-100  CPQ_11-14_ = 0-148 |  | CPQ_8-10_:  With cancer = 5.6,  Without = 8.8,  CPQ_11-14_:  With cancer = 12.5  Without = 11.8 |
| Wong[71] | 2006 | CPQ_11-14_ | 37 | Chinese | Hong Kong | Clinic |  | 29 |  |
| Wong [72] | 2011 | CPQ _11-14_- RSF:8 | 8 | Chinese | Hong Kong | School |  |  |  |
| Zhang[73] | 2007 | CPQ_11-14_ | 31 | Chinese | Hong Kong | Clinic |  | 20.1 |  |
| Zhang[74] | 2007 | CPQ_11-14_ | 37 | Chinese | Hong Kong | Clinic |  |  | Anticipation of changes following treatment = 27.4  Pre-treatment = 20.7  After 1 week of treatment = 25.6  After 1 month of treatment = 22.5  After 3 months of treatment = 21.1  After 6 months of treatment = 20.4 |
| Zhang[75] | 2008 | CPQ | 37 | Chinese | Hong Kong | Clinic |  |  | Pre-treatment = 20.7  After 1 week of treatment = 25.6  After 1 month of treatment = 22.5  After 3 months of treatment = 21.1  After 6 months of treatment = 20.4 |
| Zhang[76] | 2009 | CPQ_11-14_ | 37 | Chinese | Hong Kong | Clinic |  |  | Aesthetic component  Need = 24.2  No need = 19.7,  Dental Health Component  Need = 21.7  No need = 18.3 |

CPQ = Child Perceptions Questionnaire; ISF:8/16 = item impact version of the short form of CPQ_11-14_ (8 or 16 items respectively); RSF:8/16 = regression version of the short form of CPQ_11-14_ (8 or 16 items respectively).

TMD = temporomandibular joint dysfunction; dmfs/DMFS = decayed missing and filled surfaces (primary and permanent teeth respectively); dmft/DMFT = decayed missing and filled teeth (primary and permanent teeth respectively)DAI = Dental Aesthestic Index; IOTN = Index of Treatment Need.

1. Agou S, Locker D, Streiner DL, Tompson B: **Impact of self-esteem on the oral-health-related quality of life of children with malocclusion**. *Am J Orthod Dentofacial Orthop* 2008, **134**(4):484-489.

2. Agou S, Locker D, Muirhead V, Tompson B, Streiner DL: **Does psychological well-being influence oral-health-related quality of life reports in children receiving orthodontic treatment?** *Am J Orthod Dentofacial Orthop* 2011, **139**(3):369-377.

3. Aguilar-Diaz F-dC, Irigoyen-Camacho M-E: **Validation of the CPQ(8-10ESP) in Mexican School children in urban areas**. *Med Oral Patol Oral Cir Bucal* 2011, **16**(3):E430-E435.

4. Baker SR, Mat A, Robinson PG: **What psychosocial factors influence adolescents' oral health?** *J Dent Res* 2010, **89**(11):1230-1235.

5. Barbosa TS, Tureli MCM, Gaviao MBD: **Validity and reliability of the Child Perceptions Questionnaires applied in Brazilian children**. *BMC Oral Health* 2009, **9**:13.

6. Barbosa TS, Leme MS, Castelo PM, Gaviao MBD: **Evaluating oral health-related quality of life measure for children and preadolescents with temporomandibular disorder**. *Health Qual Life Outcomes* 2011, **9**:32.

7. Bekes K, John MT, Schaller H-G, Hirsch C: **The German version of the Child Perceptions Questionnaire on oral health-related quality of life (CPQ-G11-14) Population-based norm values**. *J Orofac Orthop* 2011, **72**(3):223-233.

8. Bendo CB, Paiva SM, Torres CS, Oliveira AC, Goursand D, Pordeus IA, Vale MP: **Association between treated/untreated traumatic dental injuries and impact on quality of life of Brazilian schoolchildren**. *Health Qual Life Outcomes* 2010, **8**:114.

9. Benson P, O'Brien C, Marshman Z: **Agreement between mothers and children with malocclusion in rating children's oral health-related quality of life**. *Am J Orthod Dentofacial Orthop* 2008, **137**(5):631-638.

10. Brown A, Al-Khayal Z: **Validity and reliability of the Arabic translation of the child oral-health-related quality of life questionnaire (CPQ11-14) in Saudi Arabia**. *Int J Paed Dent* 2006, **16**(6):405-411.

11. Cheretakis C, Locker D, Dror Y, Glogauer M: **Oral health-related quality of life of children with neutropenia**. *Spec Care Dentist* 2007, **27**(1):6-11.

12. Costa A, Ferreira MC, Serra-Negra JM, Pordeus IA, Paiva SM: **Impact of wearing fixed orthodontic appliances on oral health-related quality of life among Brazilian children**. *J Orthod* 2011, **38**(4):275-281.

13. de Oliveira CM, Sheiham A, Tsakos G, O'Brien KD: **Oral health-related quality of life and the IOTN index as predictors of children's perceived needs and acceptance for orthodontic treatment**. *Br Dent J* 2008, **204**(7):1-5.

14. Do LG, Spencer A: **Oral health-related quality of life of children by dental caries and fluorosis experience**. *J Public Health Dent* 2007, **67**(3):132-139.

15. Do LG, Spencer AJ: **Evaluation of oral health-related quality of life questionnaires in a general child population**. *Community Dent Health* 2008, **25**(4):205-210.

16. Fakhruddin KS, Lawrence HP, Kenny DJ, Locker D: **Impact of treated and untreated dental injuries on the quality of life of Ontario school children**. *Dent Traumatol* 2008, **24**(3):309-313.

17. Fergus CE: **Caries prevalence and experience of 12-year old children in Montserrat**. *West Indian Med J* 2010, **59**(5):573-577.

18. Ferreira MC, Goursand D, Bendo CB, Ramos-Jorge ML, Pordeus IA, Paiva SM: **Agreement between adolescents' and their mothers' reports of oral health-related quality of life**. *Braz Oral Res* 2012, **26**(2):112-118.

19. Foster Page LA, Thomson WM, Jokovic A, Locker D: **Validation of the Child Perceptions Questionnaire (CPQ 11-14)**. *J Dent Res* 2005, **84**(7):649-652.

20. Foster Page LA, Thomson WM, Jokovic A, Locker D: **Epidemiological evaluation of short-form versions of the Child Perception Questionnaire**. *Eur J Oral Sci* 2008, **116**(6):538-544.

21. Page LAF, Thomson WM, Mohamed AR, Traebert J: **Performance and cross-cultural comparison of the short-form version of the CPQ11-14 in New Zealand, Brunei and Brazil**. *Health Qual Life Outcomes* 2011, **9**:40.

22. Foster Page LA, Thomson WM, Ukra A, Farella M: **Factors influencing adolescents' oral health-related quality of life (OHRQoL)**. *Int J Paediatr Dent* 2013, **23**(6):415-423.

23. Foster Page LA, Thomson WM: **Caries prevalence, severity, and 3-year increment, and their impact upon New Zealand adolescents' oral-health-related quality of life**. *J Public Health Dent* 2012, **72**(4):287-294.

24. Goursand D, Paiva SM, Zarzar PM, Ramos-Jorge ML, Cornacchia GM, Pordeus IA, Allison PJ: **Cross-cultural adaptation of the Child Perceptions Questionnaire 11-14 (CPQ11-14) for the Brazilian Portuguese language**. *Health Qual Life Outcomes* 2008, **6**:2.

25. Gururatana O, Baker S, Robinson PG: **Psychometric properties of long and short forms of the Child Perceptions Questionnaire (CPQ11-14) in a Thai population**. *Community Dent Health* 2011, **28**(3):232-237.

26. Humphris G, Freeman R, Gibson B, Simpson K, Whelton H: **Oral health-related quality of life for 8-10-year-old children: an assessment of a new measure**. *Community Dent Oral Epidemiol* 2005, **33**(5):326-332.

27. Jokovic A, Locker D, Stephens M, Kenny D, Tompson B, Guyatt G: **Validity and reliability of a questionnaire for measuring child oral-health-related quality of life**. *J Dent Res* 2002, **81**(7):459-463.

28. Jokovic A, Locker D, Stephens M, Guyatt G: **Agreement between mothers and children aged 11-14 years in rating child oral health-related quality of life**. *Community Dent Oral Epidemiol* 2003, **31**(5):335-343.

29. Jokovic A, Locker D, Tompson B, Guyatt G: **Questionnaire for measuring oral health-related quality of life in eight- to ten-year-old children**. *Pediatr Dent* 2004, **26**(6):512-518.

30. Jokovic A, Locker D, Guyatt G: **What do children's global ratings of oral health and well-being measure?** *Community Dent Oral Epidemiol* 2005, **33**(3):205-211.

31. Jokovic A, Locker D, Guyatt G: **Short forms of the Child Perceptions Questionnaire for 11-14-year-old children (CPQ11-14): development and initial evaluation**. *Health Qual Life Outcomes* 2006, **4**:4.

32. Kadkhoda S, Nedjat S, Shirazi M: **Comparison of oral-health-related quality of life during treatment with headgear and functional appliances**. *Int J Paediatr Dent* 2011, **21**(5):369-373.

33. Kohli R, Levy S, Kummet CM, Dawson DV, Stanford CM: **Comparison of perceptions of oral health-related quality of life in adolescents affected with ectodermal dysplasias relative to caregivers**. *Spec Care Dentist* 2011, **31**(3):88-94.

34. Kok YV, Mageson P, Harradine NWT, Sprod AJ: **Comparing a quality of life measure and the Aesthetic Component of the Index of Orthodontic Treatment Need (IOTN) in assessing orthodontic treatment need and concern**. *J Orthod* 2004, **31**(4):312-318.

35. Kolawole KA, Otuyemi OD, Oluwadaisi AM: **Assessment of oral health-related quality of life in Nigerian children using the Child Perceptions Questionnaire (CPQ 11-14)**. *Eur J Paediatr Dent* 2011, **12**(1):55-59.

36. Koposova N, Widstrom E, Eisemann M, Koposov R, Eriksen HM: **Oral health and quality of life in Norwegian and Russian school children: A pilot study**. *Stomatologija* 2010, **12**(1):10-16.

37. Koposova N, Eriksen HM, Widstram E, Eisemann M, Opravin A, Koposov R: **Oral health-related quality of life among 12-year-olds in Northern Norway and North-West Russia**. *Oral Health Dent Manag* 2012, **11**(4):206-214.

38. Kumar S, Goyal A, Tadakamadla J, Tibdewal H, Duraiswamy P, Kulkarni S: **Oral health related quality of life among children with parents and those with no parents**. *Community Dent Health* 2011, **28**(3):227-231.

39. Laing E, Cunningham SJ, Jones S, Moles D, Gill D: **Psychosocial impact of hypodontia in children**. *Ame J Orthod Dentofacial Orthop* 2010, **137**(1):35-41.

40. Lau AWH, Wong MCM, Lam KF, McGrath C: **Confirmatory factor analysis on the health domains of the Child Perceptions Questionnaire**. *Community Dent Oral Epidemiol* 2009, **37**(2):163-170.

41. Locker D, Jokovic A, Tompson B: **Health-related quality of life of children aged 11 to 14 years with orofacial conditions**. *Cleft Palate Craniofac J* 2005, **42**(3):260-266.

42. Locker D, Jokovic A, Tompson B, Prakash P: **Is the Child Perceptions Questionnaire for 11-14 year olds sensitive to clinical and self-perceived variations in orthodontic status?** *Community Dent Oral Epidemiol* 2007, **35**(3):179-185.

43. Locker D: **Disparities in oral health-related quality of life in a population of Canadian children**. *Community Dent Oral Epidemiol* 2007, **35**(5):348-356.

44. Locker D, Jokovic A, Prakash P, Tompson B: **Oral health-related quality of life of children with oligodontia**. *Int J Paediatr Dent* 2010, **20**(1):8-14.

45. Luoto A, Lahti S, Nevanpera T, Tolvanen M, Locker D: **Oral-health-related quality of life among children with and without dental fear**. *Int J Paediatr Dent* 2009, **19**(2):115-120.

46. Marshman Z, Rodd H, Stern M, Mitchell C, Locker D, Jokovic A, Robinson PG: **An evaluation of the Child Perceptions Questionnaire in the UK**. *Community Dent Health* 2005, **22**(3):151-155.

47. Marshman Z, Gibson BJ, Benson PE: **Is the short-form Child Perceptions Questionnaire meaningful and relevant to children with malocclusion in the UK?** *J Orthod* 2010, **37**(1):29-36.

48. Martins MT, Ferreira FM, Oliveira AC, Paiva SM, Vale MP, Allison PJ, Pordeus IA: **Preliminary validation of the Brazilian version of the Child Perceptions Questionnaire 8-10**. *Eur J Paediatr Dent* 2009, **10**(3):135-140.

49. Martins-Junior PA, Marques LS, Ramos-Jorge ML: **Malocclusion: social, functional and emotional influence on children**. *J Clin Pediatr Dent* 2012, **37**(1):103-108.

50. Martins-Junior PA, Oliveira M, Marques LS, Ramos-Jorge ML: **Untreated dental caries: impact on quality of life of children of low socioeconomic status**. *Pediatr Dent* 2012, **34**(3):49-52.

51. Massarente DB, Domaneschi C, Marques HHS, Andrade SB, Goursand D, Antunes JLF: **Oral health-related quality of life of paediatric patients with AIDS**. *BMC Oral Health* 2011, **11**:2.

52. McGrath C, Pang HN, Lo ECM, King NM, Hagg U, Samman N: **Translation and evaluation of a Chinese version of the Child Oral Health-related Quality of Life measure**. *Int J Paediatr Dent* 2008, **18**(4):267-274.

53. O'Brien K, Wright JL, Conboy F, Macfarlane T, Mandall N: **The child perception questionnaire is valid for malocclusions in the United Kingdom**. *Am J Orthod Dentofacial Orthop* 2006, **129**(4):536-540.

54. O'Brien C, Benson PE, Marshman Z: **Evaluation of a quality of life measure for children with malocclusion**. *J Orthod* 2007, **34**(3):185-193.

55. Paula JS, Leite IC, Almeida AB, Ambrosano GM, Pereira AC, Mialhe FL: **The influence of oral health conditions, socioeconomic status and home environment factors on schoolchildren's self-perception of quality of life**. *Health Qual Life Outcomes* 2012, **10**:6.

56. Paula JS, Torres LH, Ambrosano GM, Mialhe FL: **Association between oral health-related quality of life and atraumatic restorative treatment in school children: an exploratory study**. *Indian J Dent Res* 2012, **23**(6):738-741.

57. Piovesan C, Ferreira Antunes JL, Guedes RS, Ardenghi TM: **Impact of socioeconomic and clinical factors on child oral health-related quality of life (COHRQoL)**. *Qual Life Res* 2010, **19**(9):1359-1366.

58. Piovesan C, Abella C, Ardenghi TM: **Child oral health-related quality of life and socioeconomic factors associated with traumatic dental injuries in schoolchildren**. *Oral health prev* 2011, **9**(4):405-411.

59. Piovesan C, Antunes JL, Mendes FM, Guedes RS, Ardenghi TM: **Influence of children's oral health-related quality of life on school performance and school absenteeism**. *J Public Health Dent* 2012, **72**(2):156-163.

60. Porritt JM, Rodd HD, Ruth Baker S: **Quality of life impacts following childhood dento-alveolar trauma**. *Dent Traumatol* 2011, **27**(1):2-9.

61. Ramos-Jorge ML, Vieira-Andrade RG, Martins-Junior PA, Cordeiro MM, Ramos-Jorge J, Paiva SM, Marques LS: **Level of agreement between self-administered and interviewer-administered CPQ(8)(-)(1)(0) and CPQ(1)(-)(1)(4)**. *Community Dent Oral Epidemiol* 2012, **40**(3):201-209.

62. Robinson PG, Nalweyiso N, Busingye J, Whitworth J: **Subjective impacts of dental caries and fluorosis in rural Ugandan children**. *Community Dent Health* 2005, **22**(4):231-236.

63. Taylor KR, Kiyak A, Huang GJ, Greenlee GM, Jolley CJ, King GJ: **Effects of malocclusion and its treatment on the quality of life of adolescents**. *Am J Orthod Dentofacial Orthop* 2009, **136**(3):382-392.

64. Torres CS, Paiva SM, Vale MP, Pordeus IA, Ramos-Jorge ML, Oliveira AC, Allison PJ: **Psychometric properties of the Brazilian version of the Child Perceptions Questionnaire (CPQ11-14) - short forms**. *Health Qual Life Outcomes* 2009, **7**:43.

65. Traebert J, de Lacerda JT, Thomson WM, Page LF, Locker D: **Differential item functioning in a Brazilian-Portuguese version of the Child Perceptions Questionnaire (CPQ(11-14))**. *Community Dent Oral Epidemiol* 2010, **38**(2):129-135.

66. Traebert J, Foster Page LA, Thomson WM, Locker D: **Differential item functioning related to ethnicity in an oral health-related quality of life measure**. *Int J Paediatr Dent* 2010, **20**(6):435-441.

67. Traebert J, Lacerda JT, Foster Page LA, Thomson WM, Bortoluzzi MC: **Impact of traumatic dental injuries on the quality of life of schoolchildren**. *Dent Traumatol* 2012, **28**(6):423-428.

68. Vargas-Ferreira F, Piovesan C, Praetzel JR, Mendes FM, Allison PJ, Ardenghi TM: **Tooth erosion with low severity does not impact child oral health-related quality of life**. *Caries Res* 2010, **44**(6):531-539.

69. Wogelius P, Gjorup H, Haubek D, Lopez R, Poulsen S: **Development of Danish version of child oral-health-related quality of life questionnaires (CPQ8-10 and CPQ11-14)**. *BMC Oral Health* 2009, **9**:11.

70. Wogelius P, Rosthoj S, Dahllof G, Poulsen S: **Oral health-related quality of life among survivors of childhood cancer**. *Int J Paediatr Dent* 2011, **21**(6):465-467.

71. Wong ATY, McMillan AS, McGrath C: **Oral health-related quality of life and severe hypodontia**. *J Oral Rehabil* 2006, **33**(12):869-873.

72. Wong MCM, Lau AWH, Lam KF, McGrath C, Lu H-X: **Assessing consistency in oral health-related quality of life (OHRQoL) across gender and stability of OHRQoL over time for adolescents using Structural Equation Modeling**. *Community Dent Oral Epidemiol* 2011, **39**(4):325-335.

73. Zhang M, McGrath C, Hagg U: **Who knows more about the impact of malocclusion on children's quality of life, mothers or fathers?** *Eur J Orthod* 2007, **29**(2):180-185.

74. Zhang M, McGrath C, Hagg U: **Patients' expectations and experiences of fixed orthodontic appliance therapy - Impact on quality of life**. *Angle Orthod* 2007, **77**(2):318-322.

75. Zhang M, McGrath C, Haegg U: **Changes in oral health-related quality of life during fixed orthodontic appliance therapy**. *Am J Orthod Dentofacial Orthop* 2008, **133**(1):25-29.

76. Zhang M, McGrath C, Hagg U: **Orthodontic treatment need and oral health-related quality among children**. *Community Dent Health* 2009, **26**(1):58-61.
