# Supplementary material for: Assessment of the quality of measures of child oral health-related quality of life
Source: BMC Oral Health. 2014 Apr 23;14:40. doi: 10.1186/1472-6831-14-40 (PMC4021173; doi:10.1186/1472-6831-14-40)
Supplement: Additional file 2 — Studies which used a version of the Child Oral Impacts on Daily Performances index with details of version, setting and range and mean scores. [file 1472-6831-14-40-S2.docx]

**Additional file 2. Studies which used a version of the Child Oral Impacts on Daily Performances index with details of version, setting and range and mean scores.**

| **Author** | **Year** | **Measure used and version** | **Number of items analysed** | **Language of version** | **Study country** | **Study population** | **Item/score distribution** | **Total mean score** | **Subgroups mean score/ proportion with impacts** |
| --- | --- | --- | --- | --- | --- | --- | --- | --- | --- |
| Bernabe[1] | 2007 | C-OIDP | 8 | Spanish | Peru | School |  | 1.3 | Proportion with impacts:  Malocclusion= 15.5% |
| Bernabe[2] | 2007 | C-OIDP | 8 | Spanish | Peru | School | 0-62.5 | 7.8 | 82% with at least one impact |
| Bernabe[3] | 2008 | C-OIDP | 8 | Spanish | Peru | School |  |  |  |
| Bernabe [4] | 2009 | C-OIDP | 8 | Thai | Thailand | School |  |  |  |
| Bianco[5] | 2010 | C-OIDP | 8 | Italian | Italy | School | 0-30 | 1.9 | 66.8% reported at least one impact |
| Castro[6] | 2008 | C-OIDP | 8 | Portuguese | Brazil | School |  | 9.2 | 80.7% with at least one impact |
| Castro[7] | 2011 | C-OIDP | 8 | Portuguese | Brazil | School |  | 7.1 | 88.7% with at least one impact  Male = 6.2  Female =7.8  DMFT>1 = 8.2  DMFT 0 = 6.3  Biofilm present =8.3  No biofilm = 6.1  Enamel defects = 9.2  No enamel defects = 7  Trauma = 7.4  No trauma = 7.1 |
| Cortes-Martinicorena[8] | 2010 | C-OIDP | 8 | Spanish | Spain | School |  |  | 11-12 years = 2.69  13-14 years = 3.08 |
| de Oliveira[9] | 2008 | CPQ _11-14_  C-OIDP | 8 | English | UK | Clinic |  |  | 49.3% reported at least one impact |
| Dumitrache [10] | 2009 | C-OIDP | 8 |  | Romania | School | Total negative effects 57.4%, |  |  |
| Gherunpong [11] | 2004 | C-OIDP | 8 | Thai | Thailand | School |  |  | Perceived treatment need = 18.1 (median)  No perceived treatment need = 5.6 (median)  Perceived oral health problems:  None/little = 5.6  Moderate = 13.9  Severe = 33.3 |
| Gherunpong [12] | 2004 | C-OIDP | 8 | Thai | Thailand | School | 0-59.7 | 8.8 | 89.8% with at least one impact |
| Gherunpong [13] | 2006 | C-OIDP | 8 | Thai | Thailand | School |  |  |  |
| Gherunpong [14] | 2006 | C-OIDP | 8 | Thai | Thailand | School |  |  | 89.8% with at least one impact  20.3% of impacts related to malocclusion |
| Krisdapong [15] | 2009 | C-OIDP | 8 | Thai | Thailand | School | 0-68.1 | 7.8 | 85.2% with at least one impact |
| Krisdapong [16] | 2012 | C-OIDP | 8 | Thai | Thailand | School |  |  | 24.7% of impacts related to recurrent apthous ulceration |
| Krisdapong [17] | 2012 | C-OIDP | 8 | Thai | Thailand | School |  |  | 26% had impacts related to calculus/gingivitis |
| Krisdapong [18] | 2012 | C-OIDP | 8 | Thai | Thailand | School |  |  | 81.7% had a least one impact |
| Krisdapong [19] | 2012 | C-OIDP | 8 | Thai | Thailand | School |  |  | 85.1% with at least one impact |
| Mashoto[20] | 2010 | C-OIDP | 8 | Kiswahili | Tanzania | School |  | T0 = 1.8  Follow up = 1 | T0:  ART = 1.3  ART and extractions = 3.9  OHE = 1.6  Follow-up:  ART = 1.5  ART and extractions = 1.7  OHE = 0.9 |
| Mbawalla [21] | 2010 | C-OIDP | 8 | Kiswahili | Tanzania | School |  |  | 48.2% with at least one impact |
| Mbawalla [22] | 2011 | C-OIDP | 8 | Kiswahili | Tanzania | School |  |  | Oral impacts:  Arusha = 0.7%  Dar es Salaam = 28.6%  Decayed teeth:  0 = 0.5  >0 = 0.8  Missing teeth:  0 = 0.6  >0 = 0.8  Oral hygiene:  Good = 0.5  Bad = 0.7  Calculus:  Good = 0.6  Poor= 0.7 |
| Mtaya[23] | 2007 | C-OIDP | 8 | Kiswahili | Tanzania | School |  | 1.2 | DMFT 0 = 1.1  DMFT >0 = 1.5 |
| Mtaya[24] | 2008 | C-OIDP | 8 | Kiswahili | Tanzania | School |  |  | 28.6% had at least one oral impact |
| Nurelhuda [25] | 2010 | C-OIDP | 8 | Arabic | Sudan | School | 0-9 | 1.5 | 54.6% had a least one impact  53.4% of those in public school  64% in private school |
| Pau[26] | 2008 | C-OIDP | 8 | English | Pakistan | School |  | 3.4 |  |
| Raymundo de Andrade[27] | 2011 | C-OIDP | 8 | Portuguese | Brazil | Clinic | 0-33 | 6.1 | Pain = 8.97  No pain = 4.4  Sensitivity = 7.65  No sensitivity = 4.48  Caries = 7.03  No caries = 5.34 |
| Rosel[28] | 2010 | C-OIDP | 8 | Spanish | Spain | School |  |  | Face to face interview 1^st^ =4.38  Face to face interview 2^nd^ = 4.21  Self-administered 1^st^ = 4.2  Self-administered 2^nd^ = 4.46 |
| Tsakos[29] | 2006 | C-OIDP | 8 | Thai | Thailand | School |  |  | Presence of impact:  Caries = 50.6%  Trauma = 4.6%  Enamel defect/anomaly = 8.7%  Malocclusion = 20.3%  Prosthodontic = 0.7% |
| Tsakos[30] | 2008 | C-OIDP | 8 | English | UK | Clinic |  |  | Mean number of impacts = 3.16 |
| Tubert Jeannin[31] | 2005 | C-OIDP | 8 | French | France | School |  | 6.3 | Not satisfied with oral health = 7.5  Satisfied with oral health = 2 Oral problems:  None = 0  Some = 4.2  Many = 19.2  No treatment need = 1 Treatment need = 5.2  Global oral health:  Very bad = 14.6  Bad = 19.2  Fairly bad = 8  Fairly good = 4.2  Good = 1.7  Excellent = 0.2 |
| Yusuf[32] | 2006 | C-OIDP | 8 | English | UK | School |  |  | 40.4% had at least one impact |
| Yusof[33] | 2012 | C-OIDP | 8 | Malay | Malaysia | School |  | 13.2 | 66.7% had at least one impact |

C-OIDP = Child Oral Impacts on Daily Performances index; dmft/DMFT = decayed missing and filled teeth (primary and permanent teeth respectively); ART = atraumatic restorative technique; OHE = oral health education; T0 = baseline measurement.

1. Bernabe E, Flores-Mir C, Sheiham A: **Prevalence, intensity and extent of Oral Impacts on Daily Performances associated with self-perceived malocclusion in 11-12-year-old children**. *BMC Oral Health* 2007, **7**:6.

2. Bernabe E, Tsakos G, Sheiham A: **Intensity and extent of oral impacts on daily performances by type of self-perceived oral problems**. *Eur J Oral Sci* 2007, **115**(2):111-116.

3. Bernabe E, Sheiham A, Tsakos G: **A comprehensive evaluation of the validity of Child-OIDP: further evidence from Peru**. *Community Dent Oral Epidemiol* 2008, **36**(4):317-325.

4. Bernabe E, Krisdapong S, Sheiham A, Tsakos G: **Comparison of the discriminative ability of the generic and condition-specific forms of the Child-OIDP index: a study on children with different types of normative dental treatment needs**. *Community Dent Oral Epidemiol* 2009, **37**(2):155-162.

5. Bianco A, Fortunato L, Nobile CGA, Pavia M: **Prevalence and determinants of oral impacts on daily performance: results from a survey among school children in Italy**. *Eur J Public Health* 2010, **20**(5):595-600.

6. Castro RAL, Cortes MIS, Leao AT, Portela MC, Souza IPR, Tsakos G, Marcenes W, Sheiham A: **Child-OIDP index in Brazil: cross-cultural adaptation and validation**. *Health Qual Life Outcomes* 2008, **6**:68.

7. Castro RdAL, Portela MC, Leao AT, de Vasconcellos MTL: **Oral health-related quality of life of 11-and 12-year-old public school children in Rio de Janeiro**. *Community Dent Oral Epidemiol* 2011, **39**(4):336-344.

8. Cortes-Martinicorena F-J, Rosel-Gallardo E, Artazcoz-Oses J, Bravo M, Tsakos G: **Adaptation and validation for Spain of the Child-Oral Impact on Daily Performance (C-OIDP) for use with adolescents**. *Med Oral Patol Oral Cir Bucal* 2010, **15**(1):e106-111.

9. de Oliveira CM, Sheiham A, Tsakos G, O'Brien KD: **Oral health-related quality of life and the IOTN index as predictors of children's perceived needs and acceptance for orthodontic treatment**. *Br Dent J* 2008, **204**(7):1-5.

10. Dumitrache MA, Comes C, Teodorescu E, Dumitrascu L, Cuculescu M, Ionescu E: **Life quality related to oral health of schoolchildren from bucharest**. *Revista Romana De Bioetica* 2009, **7**(4):169-178.

11. Gherunpong S, Tsakos G, Sheiham A: **Developing and evaluating an oral health-related quality of life index for children; the CHILD-OIDP**. *Community Dent Health* 2004, **21**(2):161-169.

12. Gherunpong S, Tsakos G, Sheiham A: **The prevalence and severity of oral impacts on daily performances in Thai primary school children**. *Health Qual Life Outcomes* 2004, **2**:57.

13. Gherunpong S, Sheiham A, Tsakos G: **A sociodental approach to assessing children's oral health needs: integrating an oral health-related quality of life (OHRQoL) measure into oral health sevice planning**. *Bull World Health Organ* 2006, **84**(1):36-42.

14. Gherunpong S, Tsakos G, Sheiham A: **A socio-dental approach to assessing children's orthodontic needs**. *Eur J Orthod* 2006, **28**(4):393-399.

15. Krisdapong S, Sheiham A, Tsakos G: **Oral health-related quality of life of 12- and 15-year-old Thai children: findings from a national survey**. *Community Dent Oral Epidemiol* 2009, **37**(6):509-517.

16. Krisdapong S, Sheiham A, Tsakos G: **Impacts of recurrent aphthous stomatitis on quality of life of 12- and 15-year-old Thai children**. *Qual Life Res* 2012, **21**(1):71-76.

17. Krisdapong S, Prasertsom P, Rattanarangsima K, Sheiham A, Tsakos G: **The impacts of gingivitis and calculus on Thai children's quality of life**. *J Clin Periodontol* 2012, **39**(9):834-843.

18. Krisdapong S, Prasertsom P, Rattanarangsima K, Sheiham A: **Relationships between oral diseases and impacts on Thai schoolchildren's quality of life: evidence from a Thai national oral health survey of 12- and 15-year-olds**. *Community Dent Oral Epidemiol* 2012, **40**(6):550-559.

19. Krisdapong S, Prasertsom P, Rattanarangsima K, Adulyanon S, Sheiham A: **Using associations between oral diseases and oral health-related quality of life in a nationally representative sample to propose oral health goals for 12-year-old children in Thailand**. *Int Dent J* 2012, **62**(6):320-330.

20. Mashoto KO, Astrom AN, Skeie MS, Masalu JR: **Changes in the quality of life of Tanzanian school children after treatment interventions using the Child-OIDP**. *Eur J Oral Sci* 2010, **118**(6):626-634.

21. Mbawalla HS, Masalu JR, Astrom AN: **Socio-demographic and behavioural correlates of oral hygiene status and oral health related quality of life, the Limpopo - Arusha school health project (LASH): A cross-sectional study**. *Bmc Pediatrics* 2010, **10**:87.

22. Mbawalla HS, Mtaya M, Masalu JR, Brudvik P, Astrom AN: **Discriminative ability of the generic and condition-specific Child-Oral Impacts on Daily Performances (Child-OIDP) by the Limpopo-Arusha School Health (LASH) Project: A cross-sectional study**. *Bmc Pediatrics* 2011, **11**:45.

23. Mtaya M, Astrom AN, Tsakos G: **Applicability of an abbreviated version of the Child-OIDP inventory among primary schoolchildren in Tanzania**. *Health Qual Life Outcomes* 2007, **5**:40.

24. Mtaya M, Astrom AN, Brudvik P: **Malocclusion, psycho-social impacts and treatment need: A cross-sectional study of Tanzanian primary school-children**. *BMC Oral Health* 2008, **8**:14.

25. Nurelhuda NM, Ahmed MF, Trovik TA, Astrom AN: **Evaluation of oral health-related quality of life among Sudanese schoolchildren using Child-OIDP inventory**. *Health Qual Life Outcomes* 2010, **8**:152.

26. Pau A, Khan SS, Babar MG, Croucher R: **Dental pain and care-seeking in 11-14-yr-old adolescents in a low-income country**. *Eur J Oral Sci* 2008, **116**(5):451-457.

27. Raymundo de Andrade LH, de Souza Rocha B, Castro GF, Ribeiro de Souza IP: **Impact of oral problems on daily activities of HIV-infected children**. *Eur J Paediatr Dent* 2011, **12**(2):75-80.

28. Rosel E, Tsakos G, Bernabe E, Sheiham A, Bravo M: **Assessing the level of agreement between the self- and interview-administered Child-OIDP**. *Community Dent Oral Epidemiol* 2010, **38**(4):340-347.

29. Tsakos G, Gherunpong S, Sheiham A: **Can oral health-related quality of life measures substitute for normative needs assessments in 11 to 12-year-old children?** *J Public Health Dent* 2006, **66**(4):263-268.

30. Tsakos G, Bernabe E, O'Brien K, Sheiham A, de Oliveira C: **Comparison of the self-administered and interviewer-administered modes of the child-OIDP**. *Health Qual Life Outcomes* 2008, **6**:40.

31. Tubert-Jeannin S, Pegon-Machat E, Gremeau-Richard C, Lecuyer M-M, Tsakos G: **Validation of a French version of the Child-OIDP index**. *Eur J Oral Sci* 2005, **113**(5):355-362.

32. Yusuf H, Gherunpong S, Sheiham A, Tsakos G: **Validation of an English version of the Child-OIDP index, an oral health-related quality of life measure for children**. *Health Qual Life Outcomes* 2006, **4**:38.

33. Yusof ZY, Jaafar N: **A Malay version of the Child Oral Impacts on Daily Performances (Child-OIDP) index: assessing validity and reliability**. *Health Qual Life Outcomes* 2012, **10**:63.
