# Supplementary material for: Assessment of the quality of measures of child oral health-related quality of life
Source: BMC Oral Health. 2014 Apr 23;14:40. doi: 10.1186/1472-6831-14-40 (PMC4021173; doi:10.1186/1472-6831-14-40)
Supplement: Additional file 3 — Studies which used a version of the Child Oral Health Impact Profile with details of version, setting and range and mean scores. [file 1472-6831-14-40-S3.docx]

**Additional file 3. Studies which used a version of the Child Oral Health Impact Profile with details of version, setting and range and mean scores.**

| **Author** | **Year** | **Measure used and version** | **Number of items analysed** | **Language of version** | **Study country** | **Study population** | **Item/score distribution** | **Total mean score** | **Subgroups mean score/ proportion with impacts** |
| --- | --- | --- | --- | --- | --- | --- | --- | --- | --- |
| Ahn[1] | 2011 | COHIP | 34 | Korean | Korea | School | 46-132 | 103.3 | Caries = 106.8  No caries = 104.2  Ortho need = 100.4  No need = 104.9 |
| Bos[2] | 2010 | COHIP | 30 | Dutch | Netherlands | Clinic |  | 112.12 | Female = 110.92  Male = 113.28 |
| Bos[3] | 2010 | COHIP | 30 | Dutch | Netherlands | Clinic |  | 119.29 | Female = 118.2  Male = 119.81  8-12 years = 121.77  >12 years = 116.19  Cleft palate = 116.29  Cleft lip = 121.65  Unilateral cleft lip and palate = 119.2  Bilateral cleft lip and palate = 119.25 |
| Broder [4] | 2007 | COHIP |  | English, French, Spanish | USA/Canada | Clinic |  |  |  |
| Broder[5] | 2007 | COHIP | 34 | English, Spanish, French | USA/Canada | Clinical/school | 28-135 | 99 | Paediatric group = 97.7  Orthodontic group = 97.2  Craniofacial group = 87.1  Community group = 102.3 |
| Broder[6] | 2012 | COHIP short form | 19 | English/Spanish | USA | Agricultural population/clinical |  |  | Caries:  COHIP = 103.5  Short form = 56.2  Malocclusion: COHIP = 102.2  Short form = 57.8, Craniofacial group:  COHIP = 98.3  Short form = 53.7 |
| Calis [7] | 2009 | COHIP | 38 | Dutch | Netherlands | School |  |  |  |
| Dunlow[8] | 2007 | COHIP | 34 | English | USA | Clinic |  |  |  |
| Geels[9] | 2008 | COHIP | 38 | Dutch | Netherlands | School |  | 1.78 |  |
| Geels[10] | 2008 | COHIP | 38 | Dutch | Netherlands | Clinic | 45-122 | 74.1 |  |
| Ravaghi[11] | 2011 | COHIP | 34 | Farsi | Iran | School | 0-82 | 28 | Perceived dental need = 32.6  No perceived need = 22.8  Pain = 35.1  No pain = 25.6  Dissatisfied with oral health = 38.5  Satisfied with oral health = 25.1  DMFT 0 = 20  DMFT >0 = 29.6  DMFT 0-2 = 25.8  DMFT >3 = 30.4 |
| Wilson-Genderson [12] | 2007 | COHIP | 34 | English | Canada/USA | Clinic |  |  |  |

COHIP = Child Oral Health Impact Profile; dmft/DMFT = decayed missing and filled teeth (primary and permanent teeth respectively).

1. Ahn YS, Kim HY, Hong SM, Patton LL, Kim JH, Noh HJ: **Validation of a Korean version of the Child Oral Health Impact Profile (COHIP) among 8- to 15-year-old school children**. *Int J Paediatr Dent* 2012, **22**(4):292-301.

2. Bos A, Hoogstraten J, Zentner A: **Perceptions of Dutch orthodontic patients and their parents on oral health-related quality of life**. *Angle Orthod* 2010, **80**(2):367-372.

3. Bos A, Prahl C: **Oral health-related quality of life in Dutch children with cleft lip and/or palate**. *Angle Orthod* 2010, **81**(5):865-871.

4. Broder HL, McGrath C, Cisneros GJ: **Questionnaire development: Face validity and item impact testing of the Child Oral Health Impact Profile**. *Community dentistry and oral epidemiology* 2007, **35**:8-19.

5. Broder HL, Wilson-Genderson M: **Reliability and convergent and discriminant validity of the Child Oral Health Impact Profile (COHIP Child's version)**. *Community Dent Oral Epidemiol* 2007, **35 Suppl 1**:20-31.

6. Broder HL, Wilson-Genderson M, Sischo L: **Reliability and validity testing for the Child Oral Health Impact Profile-Reduced (COHIP-SF 19)**. *J Public Health Dent* 2012, **72**(4):302-312.

7. Calis EM, Geels LM, Prahl-Andersen B, Zentner A: **Oral health-related quality of life and dental esthetics in Amsterdam schoolchildren**. *J Dent Child* 2009, **76**(2):130-135.

8. Dunlow N, Phillips C, Broder HL: **Concurrent validity of the COHIP**. *Community Dent Oral Epidemiol* 2007, **35**:41-49.

9. Geels LM, Hoogstraten J, Prahl-Andersen B: **Confirmative factor analysis of the dimensions of the Child Oral Health Impact Profile (Dutch version)**. *Eur J Oral Sci* 2008, **116**(2):148-152.

10. Geels LM, Kieffer JM, Hoogstraten J, Prahl-Andersen B: **Oral health-related quality of life of children with craniofacial conditions**. *Cleft Palate Craniofac J* 2008, **45**(5):461-467.

11. Ravaghi V, Ardakan MMM, Shahriari S, Mokhtari N, Underwood M: **Comparison of the COHIP and OHIP- 14 as measures of the oral health-related quality of life of adolescents**. *Community Dent Health* 2011, **28**(1):82-88.

12. Wilson-Genderson M, Broder HL, Phillips C: **Concordance between caregiver and child reports of children's oral health-related quality of life**. *Community Dent Oral Epidemiol* 2007, **35 Suppl 1**:32-40.
